# Theory for acoustic streaming in soft porous matter and its applications to ultrasound-enhanced convective delivery

**DOI:** 10.1186/s40349-018-0114-6

**Published:** 2018-08-02

**Authors:** Raghu Raghavan

**Affiliations:** Therataxis, LLC, Suite 301, 101 North Haven Street, Baltimore, 21224 MD USA

## Abstract

This paper develops theory for *bulk* acoustic streaming in soft porous materials, with applications to biological tissue. The principal results of this paper are: (i) streaming equations for such porous media, which show interestingly significant differences from those that describe streaming in pure fluids; (ii) the Green functions obtained for these equations in isotropic, infinite media; and (iii) approximate evaluation of the sources in the streaming equations from acoustic wave forms often used, and the streaming velocities and particle trajectories resulting therefrom. People are now investigating acoustic enhancement of delivery of therapeutics such as drug molecules or other particulates, introduced directly into cellular tissue. A comparison of the predictions of the theory in this paper to available data is made and shown to be surprisingly good. Some macroscale effects of the ultrastructure of the tissue that are not contained in the current paper are pointed out for future studies.

## Introduction

This paper develops the theory of bulk acoustic streaming in porous media, particularly soft materials, adapting the standard derivation of such streaming in fluids. One of our original motivations for this study was for certain applications where the transport of therapeutic molecules within tissue is assisted with the application of sonication. In general, we call the assistance of sonication for directed transport of particles *Acoustic Shepherding*. Under this rubric falls one set of applications where convection-enhanced delivery (CED) is enhanced by the application of ultrasound: we call this UeCD (ultrasound-enhanced convective delivery). CED is a standard term in the field and means the delivery of molecules in suspension into tissue by the application of a pump and UeCD is the assistance that sonication might afford for the advection of the suspended particles. (We will provide references in the more detailed discussions below). We emphasize that this is already distinct from several other applications of ultrasound in biomedicine such as destroying tissue; diagnostic ultrasound for imaging; or ultrasound for opening the blood–brain barrier for drug delivery. Rather, the purpose here is to have ultrasound (for such therapeutic applications) assist volume transmission of the drugs. The motivation for this application in turn stems from looking at the current practice in intraparenchymal infusions. This is done by inserting one or several catheters and pumping the therapeutic solution through. The tissue is a highly resistive medium for fluid flow, and there are many pathways that may lead the flow to undesired areas. Since there is only place of control where the pressure may be applied (the port of the catheter for single-port catheters, or the ports in a multiport catheter), the fluid is at the mercy of the medium to guide its path once it leaves the catheter. Sonication offers the potential to focus and direct the acoustic beams as we desire, and guide the fluid and particles to reach the target, and avoid other areas. We also mention that we expect applications of Acoustic Shepherding in other areas as well, perhaps in environmental or geophysical applications. However, the purpose of this paper is more modest, as stated above.

We develop the basic theory for bulk streaming and derive the Green functions for it. Our central simplifying homogenized medium assumption, further elucidated below, is consistent with that of the ultrasonic medical imaging community; namely that there is a longitudinal wave mode that propagates as if tissue – or the porous medium in question – were a homogeneous continuum. We ignore both the shear waves and the slow wave of Biot’s theory (we discuss the validity of these assumptions later). Following this Introduction, there are four principal sections in this paper. The goal of the “Acoustic streaming in fluids and porous media” section is to propose equations for bulk streaming and the form of the source terms therein that drive the streaming; and that of the “Green’s functions for the streaming equations” section is to write the Green function solutions for these equations (in isotropic, homogeneous, infinite porous media). We discuss in detail the simplifying assumptions made, and this necessitates some review of known material from the corresponding treatments of acoustic propagation and streaming in pure fluids. In the “Streaming in acoustic beams in homogeneous media” section, we work out the streaming source terms and the streaming velocities, for several simple acoustic sources that are often used. The “Ultrasound-enhanced convective delivery (UeCD)” section presents numerical results and also discusses experiments that have been performed on in-vivo brain tissue. In the following “Acoustic effects in structured porous media” section, we speculate on new effects that depend on the ultrastructure of cellular tissue not contained in this paper. We sum up in a concluding section. Two appendices discuss relevant material that would impede the discussion if placed in the main text.

Apart from a recapitulation of well known results in the theory of bulk streaming in fluids in the “Review of streaming in bulk fluids” subsection, the rest of the calculations and discussions are new, as far as we know. However, the principal new theoretical results are contained in the “Green’s functions for the streaming equations” section and its referents, where we summarize the streaming equations for porous media, the Green functions which also contain the pure fluid Green functions in certain limits, and a discussion of the various effects in fluid flow in porous media that we have omitted in obtaining the simplified equations. The new applications of these results are contained in the “Streaming in acoustic beams in homogeneous media” section, where the Green functions are applied to specific forms of acoustic sources, and comparison with experiments in the “Ultrasound-enhanced convective delivery (UeCD)” section.

### Prior work

We will postpone a review of prior work to two subsequent sections for the following reasons. The first is that our paper contains two somewhat separate sets of calculations: (i) the bulk streaming equations and Green’s functions for porous media which are not restricted to biological tissue; and (ii) applications of this theory to tissue and with specific acoustic sources to test if our theory can interpret some experiments on UeCD that have been performed by a variety of groups around the world. For the first, we are aware of only one such calculation to which we refer in the “Comparison with prior work” subsection. As for the second, it may be wondered why we do not review in detail the truly vast literature on acoustic effects in tissue beyond the paragraph in the “Biological effects of sonication” subsection. Again, the reason is that we have developed a theory for bulk streaming in homogenized media, and do *not* treat non-thermal streaming effects that are circulatory and depend on the presence of a nearby boundary. It is the latter that is much discussed in the literature on biological effects of ultrasound: such effects are qualitatively different from the effects described by the theory in this paper, and our discussion on them is confined to the “Acoustic effects in structured porous media” section. However, we are careful within our treatment to examine many different phenomena that could occur in porous media, even under the restrictions mentioned, to attempt to isolate the principal ones. Of course, when we venture to interpret experiments, it could be that the effects we do not develop in our theory are important ones. In the “Acoustic effects in structured porous media” section, we argue why this is unlikely to be the case. In short, the direct precursor to our basic theory is reviewed in the “Comparison with prior work” subsection, and the phenomena it focuses on in tissue are essentially independent of most of the vast literature on acoustics-tissue interactions which are briefly reviewed in the “Biological effects of sonication” subsection.

### Notation and conventions

All quantities are measured in cgs units, unless otherwise mentioned, and throughout we shall assume we are dealing with a harmonic component of an acoustic signal, i.e., the time dependence of the signal is assumed of the form *e*^−*iωt*^, where in fact the angular frequency *ω* is assumed real as well as positive. (This therefore restricts the variety of waveforms we can treat by Fourier synthesis). The symbol := or =: means that the quantity facing the colon is defined to be the quantity facing the equals sign. The unsymmetric convention is adopted for the Fourier transform, e.g.,

1 $$ \tilde{f}\left(\mathbf{k}\right) :=\int d^{3}x\exp\left(-i\mathbf{k} \cdot\mathbf{x}\right) f\left(\mathbf{x}\right)  $$

so that 1/(2*π*)^3^ occurs in the inverse, and similarly for the time – frequency transforms. Some further notation: we use ∗ to denote convolution, and ∗· to denote convolution and inner product. In other words, if *g*, **f**, and $\mathbb {H}$ are scalar, vector, and second rank tensor fields, respectively, then in Cartesian coordinates,

2 $$ {\begin{aligned} \mathbf{f}\left(\mathbf{x}\right) \mathbf{\ast}g\left(\mathbf{x}\right) &:=\int d^{3}\mathbf{x}^{\prime}\mathbf{f}\left(\mathbf{x}^{\prime}\right) g\left(\mathbf{x}-\mathbf{x}^{\prime}\right) ;\text{ \ }\left(\mathbf{f}\left(\mathbf{x}\right) \mathbf{\ast\cdot}\mathbb{H}\left(r\right) \right)_{i}\\&:=\sum_{k=x,y,z}\int d^{3}\mathbf{x}^{\prime} \mathbf{f}\left(\mathbf{x}^{\prime}\right)_{k}\cdot\mathbb{H}_{ki}\left(\mathbf{x-x}^{\prime}\right) \end{aligned}}  $$

$\mathbb {I}$ will denote the 2nd-rank identity tensor, whose Cartesian entries are the Kronecker delta *δ*_*ij*_. Also we use the usual ahistorical [1] adjectives Eulerian and Lagrangian as synonyms for the more descriptive but less used spatial and material, respectively. Our treatment will be in the Eulerian/spatial picture. Except for the “Acoustic effects in structured porous media” section, we adopt what we call the *Imaging Community* assumption: namely, the first order acoustic fields propagate as if they are longitudinal waves in a homogeneous medium (except for scattering from large inhomogeneities). This assumption has been quite successful in focusing acoustic beams even across the skull in brain [2], though in clinical application the focus must always be confirmed by magnetic resonance imaging to ensure safety. Thus there is certainly experimental authority for this assumption, which essentially is that one may regard the tissue as a medium with an effective speed of sound and density suitably averaged over the ultrastructure of the tissue. It is difficult to improve this *via*theory as is discussed in the third paragraph of the “Terms neglected” subsection. We have therefore provisionally adopted this assumption in the paper. Throughout, *c* will denote the speed of sound in the medium in question, and *δ* the Dirac delta. Citations for terms immediately available from a search on the world-wide web are often omitted.

## Acoustic streaming in fluids and porous media

In this section, we shall arrive at some equations for acoustic streaming in a porous medium which contains fluid–filled interstices. These interstices are referred to as connected pore space in the geophysical literature and as interstitial space for applications to biological tissue. We assume they fill up a volume fraction *ϕ* of the total space. If we concern ourselves with live brain tissue for example, then $\phi \gtrsim 0.2$, and the acoustic frequencies used are generally ultrasonic between 1−10 MHz. The interstitial fluid in brain flows in channels whose widths have been estimated of the order of 50 nm [3] based on diffusive transport, though taking them to be twice that on average is consistent with experiments on advective transport [4]. The skin depth is of the order of $\sqrt {\nu /\omega }$, (where *ν*$\approx 0.007\operatorname *{cm}^{2}\diagup \sec $ is the kinematic viscosity of the fluid, i.e., water at body temperature), which ranges from about 100−300 *μ* m, and is thus much larger than the channel widths. We shall therefore assume that the fluid is always in an inner boundary layer, in terms of boundary layer theory terminology.

There are at least two important differences between tissue and the usual porous medium description of rocks in geophysics. One is that the porous solid frame itself, consisting largely of cells with a weak connecting network, has about 80% of occluded fluid within it. The matter being treated is very *soft* and has even been called “liquid tissue”: see for example the index entries for this term in [5]. The second distinction is that the pore (= interstitial) space in tissue is not filled with pure fluid, but is itself a suspension which has a finite hydraulic conductivity irrespective of the cellular obstacles. The same is true of the cellular interiors. Thus we have two suspensions, separated by thin membranes connected by a weak scaffolding network. In ultrasound imaging, the speed of acoustic disturbances is taken to be close to that of water throughout, and the image is regarded as created by scattering inhomogeneities distributed within this medium. The speed of propagation is that in seawater, but the attenuation of sound is at least two of orders of magnitude larger. Our treatment below will be ‘schizophrenic’: we will treat slow fluid flow with Darcy’s law for a porous medium, but the first order acoustic wave will propagate in a uniform medium. We first review the known theory of streaming in pure fluids.

### Review of streaming in bulk fluids

The material in this section is well known, and all the equations used here are contained in Lighthill’s seminal review article [6] on streaming in bulk fluids. (Some earlier classical references on acoustic streaming are mentioned there). Our purpose in reviewing it is to establish notation that will help emphasize key differences from porous media, and also serves for us to introduce a more complete Green function for fluid streaming than is usually encountered. There are at least three streaming effects that have been examined in the past: the focus here is related to the streaming due to attenuation in bulk fluids. Another is due to phase differences (not depending on attenuation), and finally the third (historically the first to be treated), is “Rayleigh” streaming due to the presence of a boundary. We now review the first, and return to the others later.

We shall throughout assume that the fluid is at rest in the absence of the wave. In the presence of the sound wave then, the total velocity (at a spatial point **x** and at a time *t* which are both understood but suppressed as arguments for the moment):

3 $$ \mathbf{v}=:\mathbf{v}_{1}+\mathbf{v}_{2}   $$

where **v**_1_ is the first order disturbance due to the acoustic wave regarded as a perturbation, and has zero average over a cycle of a harmonic wave. We assume here for simplicity that **v**_2_, which is a period-averaged correction term second order in the magnitude of the acoustic perturbation, is *steady*. (It is not difficult to include a term allowing for transients in the streaming). Similarly, we assume the pressure has an oscillating component *p*_1_ (with zero mean over the cycle) as well as a steady second order component *p*_2_ needed to accommodate the continuity equation for the streaming velocity. The density has a resting uniform value of *ρ*_0_ and an oscillating zero mean component *ρ*_1_. Throughout this paper, we set the pressure in the absence of sound waves to zero: again, a superimposed time independent pressure gradient can be accommodated without difficulty. The density has of course a second order component which we shall assume is related by an equation of state to the pressure. We shall not need it. Then an expansion upto second order quantities results in the linearized equation for the momentum transfer due to a steady streaming velocity:

4 $$ -\eta\nabla^{2}\mathbf{v}_{2}=-\nabla p_{2}+\mathbf{f}   $$

where the right hand side is an effective body force due to the nonlinear Reynolds stress:

5 $$ \mathbf{f}=-\nabla\cdot\left(\rho_{0}\left\langle \mathbf{v}_{1} \mathbf{v}_{1}\right\rangle \right)   $$

**v**_1_**v**_1_ is a dyadic, and 〈〉 denotes averaging over a complete cycle of the harmonic wave. Lighthill refers to **f** as the forcing function for streaming arising from momentum conservation. We shall call it *streaming force*. In connection with the left hand side of (4), Lighthill remarks that it is usually incorrect to neglect the convective nonlinearity in **v**_2_ for even moderate acoustic strengths, despite the fact that it is formally a *fourth* order quantity. However, as we shall see, we will not need this term in our treatment of the porous medium, so we omit it. Define

6 $$ \mathbf{v}_{0}:=\left\langle \rho_{1}\mathbf{v}_{1}\right\rangle \diagup \rho_{0}   $$

Then, the equation of continuity averaged over a cycle is

7 $$ \nabla\cdot\mathbf{v}_{2}=-\nabla\cdot\mathbf{v}_{0}   $$

(Lighthill refers **v**_0_ as the forcing function arising from mass conservation. We shall call it *conservation force*. Rudenko [7] and others adopt the approach of defining **v**_2_ such that it is divergence-free, by adding the term $\frac {\rho _{1} \mathbf {v}_{1}}{\rho _{0}}$ into its definition. In such a case, the momentum Eq. (4) will have a different expression for the body force from that in (5). The particular arguments we make below will be affected by this transformation, but the end result will be the same).

Continuing, we also note that, at least as an approximation,

8 $$ \mathbf{v}_{0}\approx\frac{\left\langle \mathbf{I}\right\rangle }{c^{2} \rho_{0}}   $$

where **I** is the averaged intensity covector of the acoustic wave. (A fuller expression for 〈**I**〉 is in Pierce [8]). Except where stated otherwise, we will assume this approximation. Substituting (7) into the divergence applied to (4), we see that the pressure obeys

9 $$ \nabla^{2}p_{2}=\text{ }\nabla\cdot\left(\mathbf{f}-\eta\nabla^{2} \frac{\left\langle \mathbf{I}\right\rangle }{c^{2}\rho_{0}}\right)  $$

We can obtain an order of magnitude estimate for the terms within brackets on the right hand side by considering a plane wave, for example. Let the attenuation coefficient for the pressure amplitude be *β* in the direction of propagation i.e., we write *I*=*I*(*z*=0)*e*^−2*βz*^. *β* is of the order of 0.1 cm^−1^ in brain tissue at ultrasound frequencies of, say, 1.6 MHz [9], while it is only of the order of 5×10^−4^ in water at that frequency. The streaming force then has the form

10 $$ \mathbf{f}\approx2\beta\left\langle \mathbf{I}\right\rangle /c   $$

The second derivative on the right hand side of the equation generates a factor of 4*β*^2^ so that we may roughly write the right hand side as

11 $$ \frac{2\beta}{c}\left(1-\frac{2\eta\beta}{c\rho_{0}}\right) \nabla \cdot\left\langle \mathbf{I}\right\rangle  $$

The ratio

12 $$ \frac{2\eta\beta}{c\rho_{0}}:1  $$

in water, for the frequencies we consider, is less than 10^−10^ and so is usually neglected. Lighthill remarks in a footnote that we may, if we wish to correct for a small effect, compute the divergence free velocity, and add to it the term **v**_0_ which will then produce the correct divergence. We shall provide the Green function including the forcing term from mass conservation in the next section.

### Streaming in porous media

We now write down the porous medium streaming equations, in close analogy to those for pure fluids. In order to do so, we will have to neglect a number of terms. *Terms neglected below are discussed in the subsection following*. Now, the equations for a two phase porous medium are also conventionally written in a homogenized picture, which itself involves a number of assumptions, the most central one for our purposes being that the medium appears “homogenized” on the scale of a wavelength. This means that the two phases, solid and fluid, have characteristic length scales considerably smaller than a wavelength. The wavelengths in medical ultrasound approach 100 *μ* m; and the cellular obstacles are of the order of 20 *μ* m, with intercellular widths of the order of 50 nm. This continuum treatment of porous media is due to Biot [10, 11], and corrections to it from the ultrastructure of the medium are entirely beyond the scope of the paper. We begin with the standard theory of porous medium dynamics. The equation of motion of the fluid in the interstitium, and of continuity of fluid, as written for example in Coussy [12], are (*loc. cit.* Eqs. (3.39) and (1.49b))

13a $$\begin{array}{*{20}l} \rho\left(\frac{\partial\mathbf{v}}{\partial t}+\mathbf{v}\cdot \nabla\mathbf{v}\right) & =-\nabla p-\gamma\mathbf{w} \end{array} $$

13b $$\begin{array}{*{20}l} \frac{\partial}{\partial t}\left(\phi\rho\right) +\nabla\cdot\left(\phi\rho\mathbf{v}\right) & =0 \end{array} $$

13c $$\begin{array}{*{20}l} \mathbf{w} & :=\phi\left(\mathbf{v}-\mathbf{u}\right)  \end{array} $$

The actual density of the fluid is *ρ*, the interstitial fluid velocity is **v**, *γ* ≡1/*K* is the inverse of the hydraulic conductivity *K*, and **u** is the *velocity* of the porous solid frame (conventionally this symbol is reserved for the displacement). While we confine ourselves to scalar conductivities here, it is obvious how to generalize to tensors. The equation of continuity allows us to rewrite the momentum equation:

14 $$ \frac{\partial}{\partial t}\left(\phi\rho\mathbf{v}\right) +\nabla \cdot\left(\phi\rho\mathbf{vv}\right) =-\phi\left(\nabla p+\gamma \mathbf{w}\right)  $$

Now we write

15a $$\begin{array}{*{20}l} \mathbf{w} & \mathbf{=w}_{1}\mathbf{+w}_{2}+\cdots \end{array} $$

15b $$\begin{array}{*{20}l} \phi & =\phi_{0}+\phi_{1}+\cdots \end{array} $$

and so for the other dynamical variables, where the suffix indicates the order of a quantity, and all first order quantities are assumed to vanish when averaged over a cycle.

#### **Remark 1**

According to our *Imaging Community* assumption mentioned above, both *ϕ*_1_ and **w**_1_ vanish, and not just on the average. The solid (cell walls, say) and fluid are assumed here to oscillate together and so preserve the fraction of interstitial space. This is also why we do not consider fluctuations in the hydraulic resistivity or conductivity. That would contribute a term like *γ*_1_**w**_1_ in second order and would vanish without even averaging. (See also the text in the last paragraph of Appendix 1). If the walls were rigid, then **w**_1_=**v**_1_ and this second order term would have a contribution.

Expansion to second order and averaging over a cycle gives us

16 $$ {}\left\langle \phi_{0}\nabla\cdot\left(\rho_{0}\mathbf{v}_{1}\mathbf{v} _{1}\right) \right\rangle +\left\langle \phi_{1}\nabla p_{1}\right\rangle +\left\langle \gamma\phi_{1}\mathbf{w}_{1}\right\rangle =-\phi_{0}\nabla p_{2}-\gamma\phi_{0}\mathbf{w}_{2}   $$

The first term is the Reynolds stress as before. Neglecting the second and third terms on the left hand side (see below!) gives us the Eulerian velocity of streaming

17 $$ \gamma\mathbf{w}_{2}=-\nabla p_{2}-\nabla\cdot\left(\rho_{0}\left\langle \mathbf{v}_{1}\mathbf{v}_{1}\right\rangle \right) \text{ }  $$

The equation of continuity (13b) upon averaging gives

18 $$ \nabla\cdot\left(\rho_{0}\phi_{0}\mathbf{v}_{2}\right) =-\nabla \cdot\left\langle \phi_{0}\rho_{1}\mathbf{v}_{1}\right\rangle -\rho_{0} \nabla\cdot\left\langle \phi_{1}\mathbf{v}_{1}\right\rangle  $$

The solid does not stream, so that **u**_2_ is zero, and so we also have

19 $$ \nabla\cdot\left(\rho_{0}\mathbf{w}_{2}\right) =-\nabla\cdot\left\langle \phi_{0}\rho_{1}\mathbf{v}_{1}\right\rangle -\rho_{0}\nabla\cdot\left\langle \phi_{1}\mathbf{v}_{1}\right\rangle   $$

Neglecting the second term on the right hand side (again, see below), we get

20a $$\begin{array}{*{20}l} \gamma\mathbf{w}_{2} & =-\nabla p_{2}-\rho_{0}\nabla\cdot\left\langle \mathbf{v}_{1}\mathbf{v}_{1}\right\rangle  \end{array} $$

20b $$\begin{array}{*{20}l} \nabla\cdot\mathbf{w}_{2} & =-\phi_{0}\frac{\nabla\cdot\left\langle \rho_{1}\mathbf{v}_{1}\right\rangle }{\rho_{0}}  \end{array} $$

This has the form of Darcy’s law, now supplemented by a streaming force term, as we shall call the second term on the right hand side of (20a), together with an equation of continuity for the fluid, which *is* compressible. There is of course a perhaps unconscionable number of terms neglected in arriving at these equations, which we will discuss below. However, we *cannot* neglect the divergence of the streaming velocity. Taking the divergence of Eq. (20a), and substituting (20b), we find

21 $$ \nabla^{2}p_{2}=\nabla\cdot\left(\mathbf{f}+\frac{\gamma\phi_{0}}{\rho_{0} }\left\langle \rho_{1}\mathbf{v}_{1}\right\rangle \right)  $$

with

22 $$ \mathbf{f}:=-\rho_{0}\nabla\cdot\left\langle \mathbf{v}_{1}\mathbf{v} _{1}\right\rangle   $$

For a porous medium we restrict **I** (and **f**) to be those quantities arising only from the standard sound wave in tissue. Let us therefore assume that the approximations (8) along with the definition (6) and (10) hold for acoustics in porous media as well. Then, by the same argument as in the previous subsection for a plane wave, we see that the ratio of the magnitude of the second term within the parentheses on the right to that of the first is of the order of

23 $$ \frac{\phi_{0}}{2K\beta c\rho_{0}}:1  $$

where *K* is the magnitude of the hydraulic conductivity. We see now that the ratio of the two factors instead of being 10^−7^:1 as for a pure fluid, (if we introduce the higher attenuation coefficient appropriate to tissue into equation (12)) is of the order of 100:1 for brain tissue, and dominates the other term. Another way of saying the latter is that **v**_0_ in (6) is much smaller than the total streaming velocity in a pure fluid and hence may be neglected there, but not in a porous medium, where it is dominant. We will confirm this order of magnitude argument on the relative ineffectiveness of the streaming force, and of the importance of the conservation force, with detailed calculations below.

Thus, an approximate equation for the isothermal mean Eulerian streaming velocity due to acoustic sources in a passive porous medium is (20a), supplemented by the equation of continuity (20b). The right hand sides of these equations need separate evaluation from a knowledge of the acoustic sources and the linear wave propagation characteristics in the medium.

### Terms neglected

We now discuss several of the neglected terms. Even in an isotropic homogeneous porous medium, as Biot showed, [10, 11] there are three acoustic modes: known as fast, slow, and shear. The slow mode for such soft materials as gels or tissue is largely diffusive at even the highest frequencies we currently envisage, and damps quickly within the medium [13, 14]. The shear wave propagates only in the solid framework. Moreover it is very highly damped (for the frequencies we consider), and its wavelengths then are so small such that Biot’s theory itself is not be a good approximation. For the bulk or volume effects treated in this paper, we neglect this.

Continuing, Eq. (13a) neglects the inertial drag term, as Coussy points out, or what is called the “dynamic tortuosity”, which couples the fluid inertia to that of the solid. This results in an acceleration that must be added to the left hand side of that equation within the brackets of the form [12]

24 $$ \mathbf{a}=\left(a-1\right) \left(\mathbf{a}^{f}-\mathbf{a} ^{s}-\mathbf{w}\frac{\nabla\cdot\mathbf{u}}{\phi}\right)  $$

where *a*>1 is a number, and **a**^*f*^,**a**^*s*^ are the accelerations of fluid and solid, which include the convective term since these are Eulerian quantities. Now, to first order in |**v**|/*c*, these are indeed important in determining the dispersion relation of the acoustic waves. However, we neglect their second order contribution. Next, there is yet a further addition to, say the right hand side of (20a), neglected by Coussy, that arises from a more careful treatment of averaging the Navier Stokes equation in a porous medium. In form given by Whittaker [15] (see his Eq. (116)), it is

25 $$ -\frac{\rho_{0}}{\phi}\nabla\cdot\left(\mathbf{v}_{1}\mathbb{B} \mathbf{v}_{1}\right)  $$

where $\mathbb {B}$ is a dimensionless, positive definite matrix of the order of unity. Its spatial scale is that of the interstitial widths (for which, see Introduction section). A first principles evaluation of this term would depend on the microscopic model assumed for the porous medium. Whitaker in another paper [16] — see his equation (C.10) there — evaluates this for a bundle of capillary tubes, for which its average reduces to 1/4 of the streaming force we use in (20a), and in the same direction, thus enhancing this force. We do not specifically account for this term: it merely introduces a further numerical uncertainty which is relatively small in comparison with all the other approximations (and because the streaming force will turn out to be unimportant).

Now we turn to the terms already discussed, namely 〈*ϕ*_1_∇*p*_1_〉,〈*γϕ*_1_**w**_1_〉, and 〈*ϕ*_1_**v**_1_〉. It may be thought that the Biot theory of acoustics in porous media would suffice to evaluate these terms, particularly as it has been shown to work well for soft matter such as gels [13]. However, the theory is quite unstable for such soft porous materials. It is easy to see why, for example by using the formulas given in the review paper by Pride [14]. In particular Eq. (9.21) of Pride’s article is an explicit expression for the ratio of the amplitudes **w**_1_ and **u**_1_. (Pride uses the letters to mean the displacement amplitudes, while we mean the velocity amplitudes, but this makes no difference for the ratio of the two in a harmonic wave). In *cgs* units, both numerator and denominator in his expressions involve subtracting (about) 1 from a product of two numbers, one of which is of the order of 10^10^ and the other of the order of 10^−10^. It seems hopeless to compute all the numbers from first principles and get even the sign correct. The imaging literature essentially bypasses the Biot theory and assumes a single velocity of sound as in a homogeneous medium. This is tantamount to assuming **w**_1_=0, in which case it is easy to justify ignoring the above terms. We therefore accept the simplifications introduced, and defer a more rigorous study of them to the future. The equations we have are a natural extension of Darcy’s law, with the change in the continuity equation as we have pointed out.

Next, the usual arguments suffice for us to neglect the bulk velocity term of the form *η*∇^2^**w**_2_ (known as the Brinkman term) in the porous streaming equations. This also applies to any convective nonlinearities of the form (**w**_2_·∇)**w**_2_ although these are the terms, as Lighthill mentions, that dominate streaming in pure fluids at any but the weakest acoustic intensities. We can see that if *L* is the characteristic length scale for variation of **w**_2_, the relative ratios of

26 $$ {}\gamma\mathbf{w}_{2},\rho\left(\mathbf{w}_{2}\cdot\nabla\right) \mathbf{w}_{2},\eta\nabla^{2}\mathbf{w}_{2}\text{ are those of }\gamma,\rho\mathbf{w}_{2}/L,\eta/L^{2}\text{,}  $$

respectively, in terms of order of magnitude. (A term *ζ*∇(∇·**w**_2_) where *ζ* is the second viscosity will be even more negligible than the Brinkman term). An upper estimate of the relative importance of the terms neglected is obtained by choosing a lower limit for the length scale and an upper limit for the velocity. For the length scale, we take to be the lowest wavelengths we consider: this is of the order of 1/10 mm. Choose further a hydraulic conductivity of tissue no larger than 10^−6^ (which is quite high for tissue), and a viscosity of the order of 0.01. The amplitude of the velocity due to an acoustic beam is of the order of *p*/*ρ*_0_*c* where *p* is the pressure amplitude. (This is far larger than any streaming velocity so we are erring on the side of caution). One cannot apply more than 10 atmospheres of pressure, and so the velocity amplitudes are no larger than about 10^7^/10^5^=10^2^. This gives the ratios in the form

27 $$ 10^{6}:10^{4}:10^{2}  $$

where we have been extremely generous in allowing for the importance of the non-Darcy terms. It is seen that the the Brinkman term is indeed entirely negligible. The convective nonlinearity has been grossly overestimated but is still a small effect. The other side of this coin is that a pure fluid, where the Darcy term is absent, will respond far more strongly to body forces (the responses are in inverse proportions to the above coefficients). In fact, without solving for the velocities, and by regarding the effect of the Laplacian as being of the order of 1/*L*^2^, we may arrive at a naive estimate of the ratio of the streaming velocities in the two cases as very crudely

28 $$ {}\mathbf{v}_{\operatorname*{liquid}}:\mathbf{v}_{\operatorname*{porous}} \!\sim\!\left(\!\eta\nabla^{2}\!\right)^{-1}\!:\!\gamma\!^{-1}\!\gtrsim\!10^{-4} /10^{-2}:\!10^{-7}\!\approx10^{5}:1  $$

Detailed calculations below show that this is indeed the case.

Finally, in living brain tissue, there is a net influx of fluid into most regions of tissue coming from the capillaries, which form the source that replaces interstitial fluid absorbed into the cerebrospinal fluid. In a normal brain, this is of the order of 0.1−0.4 *μ* L/ min/ gm of brain tissue [17]. In a diseased state such as primary brain cancer, there is an additional influx at a rate about ten times higher from the tumor, i.e., at about 2 *μ* L/ min/ gm of tumor. The brain is of the order of 1500 gms, and that even relatively large primary brain tumors will not exceed 100 gms. Approximating the tissue density to be about 1 gm/ cc, this means an additional term of the order of 3×10^−6^ in intact tissue (and a term ten times larger in tumor interstitium) which belong to the right hand of Eq. (20b). On the other hand, by substituting useful values for acoustic intensities and the speed of sound, we find the retained term is of the order of 10^−4^. Thus, the endogenous interstitial flow is indeed negligible and the velocities of the interstitial fluid even more so, except perhaps close to the ventricular and cerebrospinal fluid (CSF) sinks. In any case, a modified form for the continuity equation in brain tissue would be:

29 $$ \nabla\cdot\mathbf{w}_{2}=-\phi_{0}\frac{\nabla\cdot\left\langle \rho_{1}\mathbf{v}_{1}\right\rangle }{\rho_{0}}+q_{i}   $$

where *q*_*i*_ (*i* for “intrinsic”) must be obtained from external considerations and knowledge of tissue properties. This additional steady source of fluid will of course affect the resting steady pressure *p*_2_ in the medium. As before, 〈*ρ*_1_**v**_1_〉 is computed from first order acoustics.

### Particle trajectories

In the preceding, we have identified what is known as the “mean Eulerian velocity” of streaming. It is a vector field in space. If it were truly the mean velocity of a particle situated at that point, we would simply integrate it to obtain the trajectory of the particle released at a certain point. However, as has been well known for a long time, this is not the case. Andrews and McIntyre [18] constructed the appropriate general theory to deal with such velocities for which they use the adjective *Lagrangian-mean*, but we have not yet exploited their formalism. Secondly, as noted by Westervelt [19] decades prior to Lighthill’s article, it really should be **v**+**v**_Stokes_ that should be regarded as this Lagrangian-mean velocity, where **v**_Stokes_ is defined in Appendix 1. It is true that **v**_Stokes_ and **v**_0_ have the same divergence, but they need not be irrotational, and so may have different curls. It should be mentioned that this addition mentioned by Lighthill is quite distinct from his suggestion quoted at the end of the “Review of streaming in bulk fluids” subsection of the present paper to add **v**_0_ to the mean *Eulerian* velocity, when the divergence of the streaming velocity as given by (7) is ignored! Finally, and most importantly for our purposes, his remarks pertain to pure fluids which were the subject of his talk and his paper based on it. In Appendix 1, we consider Stokes drift in detail and argue that it should be neglected for porous materials such as those in which we are interested. We therefore ignore this difference.

### Comparison with prior work

There is a vast amount of work on streaming and other acoustic effects in biological tissue which we will mention in the “Acoustic effects in structured porous media” section. However, we are aware of only the work of Poesio [20] and his colleagues that directly relates to our treatment, where they investigate a one–dimensional model, for applications to geophysics. They make the conventional assumption that the streaming velocity is divergence–free. This also immediately results, in their one-dimensional model, in the fact that any steady streaming velocity must be constant, so that any non-constant pressure gradient serves simply to counterbalance the streaming force. Even including the neglected divergence of the streaming velocity, with all quantities have only an *x*-dependence, we have

30a $$\begin{array}{*{20}l} K^{-1}w & =-dp\diagup dx+\frac{\beta}{c}I_{0}e^{-\beta x} \end{array} $$

30b $$\begin{array}{*{20}l} \frac{dw}{dx} & =\frac{\beta I_{0}}{\rho_{0}c^{2}}e^{-\beta x} \end{array} $$

in obvious notation. These equations for speed and pressure are obviously integrable directly: the velocity is an *increasing* function of distance from the acoustic source, and, depending on the boundary conditions applied, can be negative, counterstreaming to the source. When the right hand side of the second equation is set to zero, as they do, we have immediately that *w* must be constant. The effects are essentially trivial in the one–dimensional case. Secondly Poesio et. al. include both a fast and a slow wave in computing the streaming force: for soft materials at any rate the slow wave is damped too fast to matter. A further problem is that the slow wave must be separately treated: the solid and fluid are opposed in phase, and it can be seen from our treatment above that neglecting the other terms that arise in this case will require separate justification at the least, and it may in fact be invalid to do so. Finally, they compute the attenuation from Biot’s theory. At least for soft matter, this would result in a serious underestimate (by two orders of magnitude) in the attenuation coefficients which drive streaming. It is the *bulk*viscosity that drives attenuation of the acoustic waves in such media, even though we can of course entirely neglect bulk viscosity (the Brinkman term) in the streaming equation. Our treatment is three-dimensional and includes the non-negligible conservation force.

## Green’s functions for the streaming equations

We shall compute the Green functions for streaming in an infinite, homogeneous medium with no boundaries. *Henceforth, we adopt the following notation.* **v**,*p* (instead of **w**_2_,*p*_2_) will now denote the second order steady state streaming velocity and pressure, respectively. We re-introduce the Brinkman term, though, and write

31a $$\begin{array}{*{20}l} \gamma\mathbf{v} & =\eta\nabla^{2}\mathbf{v}-\nabla p+\mathbf{f}  \end{array} $$

31b $$\begin{array}{*{20}l} \mathbf{\nabla\cdot v} & =-\mathbf{\nabla\cdot v}_{0}=:q_{0}  \end{array} $$

into the streaming equations for a porous medium. The reason for the re-introduction of the Brinkman term is that we can exhibit the Green functions for streaming in both pure fluids and for porous media by taking suitable limits of the Green function covering both theories. This demands that we retain the bulk viscosity term in addition to the streaming for a medium described by Darcy’s law. From (20b), we see that

32 $$ \mathbf{v}_{0}:=\phi_{0}\frac{\left\langle \rho_{1}\mathbf{v}_{1}\right\rangle }{\rho_{0}}  $$

provided, as we shall assume, that *ϕ*_0_,*ρ*_0_ have no spatial variation. (For a pure fluid, *ϕ*_0_=1). If there are endogenous sources, these must be added to **v**_0_ as discussed above. The streaming force **f** – see (22) – and the ‘velocity’ **v**_0_ are assumed to be prescribed from a knowledge of the first order acoustic wave in the tissue for the purposes of exhibiting the Green functions.

We solve for the pressure. Using (31b) in the divergence operator applied to (31a) we get

33 $$ \nabla^{2}p=-\eta\nabla^{2}\mathbf{\nabla\cdot v}_{0}+\gamma\mathbf{\nabla \cdot v}_{0}+\mathbf{\nabla\cdot f}  $$

Since the right hand side is assumed known, the pressure is obtained as

34 $$ p=-\frac{1}{4\pi}\left(\left[ \gamma-\eta\nabla^{2}\right] \mathbf{\nabla \cdot v}_{0}+\mathbf{\nabla\cdot f}\right) \ast\frac{1}{r}   $$

This is not yet in the usual form which involves a convolution directly with the forcing terms **f**,**v**_0_. Before we proceed to do that, we note that for many computational purposes, particularly when the divergences are available in simple form, (34) is the most convenient and stable equation to use to solve for the pressure. The reason is that in using (34), we can control the approximations we make for the first order acoustic disturbances so that these sources strictly vanish in the absence of attenuation, as they should. When we write the solution as operations on **f**, **v**_0_, then for complicated acoustic fields in tissue it is very easy to make approximations that do not result in energy conservation in the absence of attenuation, and the results then become obviously incorrect. If all the calculations are numerical, then it may be advantageous to use the forms below, since integrals are more numerically stable than derivatives. However, in *our* numerical calculations in the next section, we have used the form given in (34). Further remarks on the two forms are given below in the section on spherical sources.

In Appendix 2, we indicate how to obtain the required Green function. There we find the velocity to be given by equation (132), reproduced here:

35 $$ {}\begin{aligned}4\pi\gamma\mathbf{v}=\gamma\mathbf{v}_{0}\ast\cdot\text{ }\nabla\nabla\frac {1}{r}+\mathbf{f}\ast\cdot\text{ }\nabla\nabla\frac{1-\exp\left(-\alpha r\right) }{r}+\alpha^{2}\mathbf{f}\ast\exp\left(-\alpha r\right) /r  \end{aligned}  $$

To make this more explicit, we use the fact that [21]

36 $$ \nabla\nabla\frac{1}{r}=\frac{3\hat{\mathbf{x}}\hat{\mathbf{x}}-\mathbb{I}}{r^{3}} -4\pi\hat{\mathbf{x}}\hat{\mathbf{x}}\delta^{\left(3\right) }\left(\mathbf{x} \right) =:\mathbb{Q-S}   $$

where $\mathbb {Q},\mathbb {S}$ identify the regular and the singular (distributional) parts of the dyadic tensor^1^. In Cartesian coordinates,

37 $$ \frac{\partial^{2}}{\partial x_{i}\partial x_{j}}\frac{1}{r}=\frac{3x_{i} x_{j}-r^{2}\delta_{ij}}{r^{5}}-4\pi\frac{x_{i}x_{j}}{r^{2}}\delta^{\left(3\right) }\left(\mathbf{x}\right)   $$

Applying this to 1/*r* and to (1− exp(−*αr*))/*r* in (132) and collecting terms finally yields

38 $$\begin{array}{*{20}l} 4\pi\gamma\mathbf{v} & =\mathbf{f}\ast\cdot\left\{ \left[ 1-\exp\left(-\alpha r\right) -\alpha r\exp\left(-\alpha r\right) \right] \mathbb{Q}\right\} \\ & +\alpha^{2}\mathbf{f}\ast\cdot\left\{ \left(\mathbb{I}-\hat{\mathbf{x}}\hat{\mathbf{x}}\right) \exp\left(-\alpha r\right) /r\right\} \\ & +\gamma\mathbf{v}_{0}\left(\mathbf{r}\right) \ast\mathbf{\cdot}\text{ }\left(\mathbb{Q-S}\right)  \end{array} $$

During the calculation, there will occur a term involving a convolution of **f** with the singular delta distribution which has a factor (1− exp(−*αr*)). Both terms are regular at the origin and they cancel for finite *α*, so we have omitted it with the result that only **v**_0_ remains to be evaluated at the origin. Equation (38) together with the definitions of the dyadic tensors in (36) exhibits the Green tensor for the velocity field to be convolved with the sources **f**, **v**_0_.

The porous medium limit requires *α*→*∞*, and this limit is evaluated in Appendix 2. However, we can derive the porous medium equations more directly from (34) by discarding the Brinkman term to write

39 $$ p=-\frac{1}{4\pi}\left\{ \mathbf{\nabla\cdot}\left(\mathbf{f+} \gamma\mathbf{v}_{0}\right) \right\} \ast\frac{1}{r}=-\frac{1}{4\pi}\left(\mathbf{f+}\gamma\mathbf{v}_{0}\right) \ast\cdot\nabla\frac{1}{r}   $$

The velocity

40 $$ \gamma\mathbf{v}=-\nabla p+\mathbf{f}   $$

and is therefore given by

41 $$ \gamma\mathbf{v=}\frac{1}{4\pi}\left(\mathbf{f+}\gamma\mathbf{v}_{0}\right) \ast\cdot\nabla\nabla\frac{1}{r}+\mathbf{f}   $$

with the dyadic given by (36) above.

In the form (41) written, the streaming velocity is computed as the sum of three terms, one due to the compressibility of the fluid (indicated by **v**_0_) and two due to the streaming force. For a fixed sound speed, the term due to **v**_0_ is proportional to the interstitial volume fraction, while those due to the streaming force are proportional to both the attenuation coefficient as well as the hydraulic conductivity (and hence to the interstitial fraction, but implicitly). All terms are proportional to the power of the acoustic source. Often, the two contributions from the streaming force cancel with each other.

## Streaming in acoustic beams in homogeneous media

In this section, we assume some very simple acoustic patterns to show the order of magnitude of the streaming speeds obtained in our theory. *We will simply use the bulk medium approximations*

42a $$\begin{array}{*{20}l} \mathbf{f} & =2\beta\left\vert \left\langle \mathbf{I}\right\rangle \right\vert /c \end{array} $$

42b $$\begin{array}{*{20}l} \mathbf{v}_{0} & =\phi_{0}\left\vert \left\langle \mathbf{I}\right\rangle \right\vert /\rho_{0}c^{2}  \end{array} $$

(These approximations are true for simple wavefronts such as plane waves, with small attenuation coefficients. We have not ascertained the quality of these approximations from the point of view of the Biot theory for reasons alluded to earlier in the “Terms neglected” subsection). We then see that the fluid pressure in a porous medium without boundaries, Eq. (39) becomes

43a $$\begin{array}{*{20}l} p & =\frac{1}{4\pi\mathcal{D}}q\ast\frac{1}{r} \end{array} $$

43b $$\begin{array}{*{20}l} q & =-\nabla\cdot\left\langle \mathbf{I}\right\rangle  \end{array} $$

43c $$\begin{array}{*{20}l} \mathcal{D}^{-1}\text{} & \mathcal{=}\frac{2\beta}{c}+\frac{\phi_{0}\gamma }{\rho_{0}c^{2}}  \end{array} $$

Although the second term in $\mathcal {D}^{-1}$ completely dominates the first (as discussed extensively before where we have pointed out that the opposite is true for pure fluids), we shall retain both because we will see exact cancellation of the first term in certain cases. The models we compute will all have streaming velocities dependent on only one linear dimension, say a radial one *r*. Denote the streaming velocity as a function of one variable as *v*(*r*), where this is either a radial speed in a spherically symmetric case, or a linear speed in a paraxial approximation. Then, the time taken to reach a given distance *R* starting from *ε* will be computed by

44 $$ T\left(R\right) =\phi\int_{\varepsilon}^{R}\frac{dr}{v\left(r\right) }   $$

The appearance of the interstitial volume fraction *ϕ* is because we have been computing the Darcy velocity which is a factor of *ϕ* smaller than the interstitial velocity (see Appendix 1 of [25] for an elaboration). For the simple cases we treat below, we can use these convolutions (Green functions) to obtain the desired results, but we have to accommodate boundary conditions by adding a solution to the Laplace equation with Neumann (reflecting) boundary conditions. In other words, we add a potential *Ψ* such that

45 $$\begin{array}{*{20}l} \gamma\mathbf{v} & =-\nabla p-\nabla\Psi+\mathbf{f} \end{array} $$

46 $$\begin{array}{*{20}l} \nabla^{2}\Psi & =0\text{ }\&\text{ }\left. \mathbf{v}\right\vert_{\mathcal{B}}=0  \end{array} $$

with *p* given by (43a), i.e., the Green function solution. The notation $\left. \mathbf {v}\right \vert _{\mathcal {B}}$ means the right hand side of (45) restricted to the boundary. The boundaries will be specified below case-by-case.

### Acoustic sources

We now discuss some acoustic sources to obtain the streaming force **f** and the conservation force **v**_0_. As is well known, e.g., in [26], diffraction effects make the amplitude and intensity patterns from simple geometric sources quite complex even in simplified approximations. “The difficulty of solving [the equations for streaming] for any but the simplest acoustic field and the simplest geometry is obvious” [27], and so we confine ourselves to crude approximations in the hope that the calculations yield at least the right order of magnitude. We discuss the different beam patterns we use, and compute the streaming velocities from the Green functions. We also add the boundary conditions that may be appropriate to see how the results are modified (cancellations ensue). Comparisions with what people have observed in experiments aimed at enhancing drug delivery to the brain is postponed to the following section. Apart from the pulsating spherical source pattern which is included because it is instructive and serves to illustrate the point made at the end of the previous footnote, the other sources are included because they have been, or are approximations to, the sources used in the experiments we shall examine.

#### Spherical pulsations

We consider the form of the spherical source to be

47a $$ \left\langle \mathbf{I}\right\rangle =\frac{P}{4\pi}\exp\left(-2\beta r\right) /r^{2}\hat{\mathbf{r}}   $$

so that

47b $$ \nabla\cdot\left\langle \mathbf{I}\right\rangle =-2\beta\left\vert \left\langle \mathbf{I}\right\rangle \right\vert +P\delta^{3}\left(\mathbf{x}\right)   $$

We compute first the convolution of the first term (in accordance with Eq. (43a):

48 $$ p_{1}=\frac{P}{4\pi\mathcal{D}}\frac{\beta}{2\pi}\left(\frac{\exp\left(-2\beta r\right) }{r^{2}}\mathbf{\ast}\frac{1}{r}\right)  $$

We may compute this directly using spherical polar coordinates; or by using the spherical expansion for the Laplace kernel [22],

49 $$ \frac{1}{\left\vert \mathbf{x}-\mathbf{x}^{\prime}\right\vert }=\sum_{l=0}^{\infty}\frac{\left(\min\left(r,r^{\prime}\right) \right)^{l} }{\left(\max\left(r,r^{\prime}\right) \right)^{l+1}}P_{l}\left(\zeta\right)  $$

where *ζ* is the cosine of the angle between (**x**,**x**^′^), and *P*_*l*_ the customary symbol for the Legendre polynomials, as well as

50 $$ \int_{-1}^{1}d\zeta^{\prime}P_{l}\left(\zeta^{\prime}\right) =2\delta_{l,0}  $$

In either case, we obtain

51 $$ -\nabla p_{1}=\frac{P}{4\pi\mathcal{D}}\frac{1-\exp\left(-2\beta r\right) }{r^{2}}\hat{\mathbf{r}}   $$

However, the convolution with the delta function (the second term in (47b)) gives, for the negative gradient of the contribution to the pressure from this term,

52 $$ -\nabla p_{2}=-\frac{P}{4\pi\mathcal{D}}\frac{1}{r^{2}}\hat{\mathbf{r}}   $$

The contribution of the streaming force, namely the last term in (45), to the streaming velocity is from (42a), using (47a),

53 $$ \gamma\mathbf{v}_{3}=\frac{2\beta}{c}\left\langle \mathbf{I}\right\rangle =\frac{2\beta}{c}\frac{P}{4\pi}\exp\left(-2\beta r\right) /r^{2} \hat{\mathbf{r}}   $$

We add (51), (52), and (53). Taking note of the definitions (43c) as well as (42b), we find two cancellations, and we are left with our estimate of the streaming velocity being

54 $$ \mathbf{v}_{S}\left(r\right) =-\mathbf{v}_{0}\left(r\right)   $$

which is negative *inward*. Let us now correct this calculation by adding a boundary condition. We assume that there is a spherical radiator of radius *ε*, say, and add the potential *Ψ* which is harmonic outside this sphere, for radius *r*>*ε* and is adjusted to prevent any streaming at the surface of this source which is considered an impermeable surface within the medium. In fact we can take

55 $$ \Psi=\frac{A}{4\pi\mathcal{D}}\frac{1}{r}  $$

We adjust *A* to cancel the flux coming from *p*_2_ and from the streaming force term (42a). The delta function contribution can be ignored since the origin is outside the region of integration, and the result is

56 $$ \mathbf{v}_{S}\left(r\right) =P\frac{\phi_{0}}{\rho_{0}c^{2}}\frac {\exp\left(-2\beta\varepsilon\right) -\exp\left(-2\beta r\right) }{4\pi r^{2}}  $$

entirely independent of the hydraulic conductivity, and of the streaming force, the contribution of which canceled with that of the harmonic potential required to ensure vanishing streaming at the physical boundary of the radiator. The above solution pertains of course only for *r*>*ε*, and is always positive (zero at the surface) and for *βr*>>1 is dominated by the first term.

##### The Green function singularity

The forcing terms in this example are singular at the origin and serve to illustrate the point made in the previous endnote. If we were to use the isotropic form − 4*πδ*^(3)^(**x**)/3 for the distributional portion $\mathbb {S}$ in Eq. (36) of the Green function, we would obtain 1/3 of the expression (54). A careful treatment where the limiting forms as the radial coordinate *r*→0 are taken first, i.e., before application of the Dirac delta, using the actually given form for $\mathbb {S}$ defined in Eq. (36) given in explicit form in equation (37), yields results in agreement with the simpler calculation given above. In such calculations, the convolution with the regular part $\mathbb {Q}$ of the dyadic may encounter a singular integrand: that integral then must, as usual, be evaluated as a principal value. In this example, the convolution with $\mathbb {Q}$ vanishes.

#### Cylinder with radial pulsations

As a second example, let us take an infinite cylindrical source pulsating radially. We approximate this as

57a $$\begin{array}{*{20}l} \left\langle \mathbf{I}\right\rangle & =\frac{P}{2\pi}\frac{\exp\left(-2\beta\rho\right) }{\rho}\hat{\mathbf{\rho}} \end{array} $$

57b $$\begin{array}{*{20}l} \nabla\cdot\left\langle \mathbf{I}\right\rangle & =-2\beta\left\vert \left\langle \mathbf{I}\right\rangle \right\vert  \end{array} $$

outside the origin. The expression (57a) is exact for the period-averaged intensity of an infinite cylindrical source and is a consequence of Hankel form for the velocity potential of a propagating wave from the infinite cylinder [28], and the Wronskian identity

58 $$ J_{0}\left(z\right) Y_{1}\left(z\right) -J_{1}\left(z\right) Y_{0}\left(z\right) =2/\left(\pi z\right)  $$

*P* of course has a different meaning and unit than for the spherical source: it is now the power radiated per unit length of the cylindrical source. We shall again consider the surface of such a radiator to be at *ρ*=*ε* while the source of the fluid is at *ρ*=*a*>*ε*. Such sources (though of course not uniform infinite cylinders) do exist, and we shall compare with experiment in the next section. This gives

59 $$ p_{2}=\frac{P}{2\pi}\left(\frac{\phi_{0}\gamma}{\rho_{0}c^{2}}+\frac{2\beta }{c}\right) \frac{2\beta}{2\pi}\left(\frac{\exp\left(-2\beta\rho\right) }{\rho}\mathbf{\ast}\ln\left(1/\rho\right) \right)   $$

upon using the two dimensional Green function ln(1/*ρ*) satisfying

60 $$ \nabla^{2}\ln\left(1/\rho\right) =-2\pi\delta^{2}\left(\mathbf{x}\right)  $$

∇^2^ now being the *two-dimensional* Laplacian. Defining

61 $$\begin{array}{*{20}l} \rho_{<} & :=\min\left(\rho,\rho^{\prime}\right) \end{array} $$

62 $$\begin{array}{*{20}l} \rho_{>} & :=\max\left(\rho,\rho^{\prime}\right) \end{array} $$

we use now the cylindrical expansion for the Coulomb kernel [22]:

63 $$ {}\begin{aligned} \ln\left(\frac{1}{\sqrt{\rho^{2}+\rho^{\prime2}-2\rho\rho^{\prime}\cos [\phi-\phi^{\prime}]}}\right) & =\ln\left(1/\rho_{>}\right) +\\ & \sum_{m=1}^{\infty}\frac{1}{m}\frac{\rho_{<}}{\rho_{>}}\cos\left(m[\phi-\phi^{\prime}]\right) \end{aligned}  $$

The integration over *ϕ*^′^ disposes of the terms in the sum, and we are left with

64 $$ -\nabla p_{2}=\frac{P}{2\pi}\left(\frac{\phi_{0}\gamma}{\rho_{0}c^{2}} +\frac{2\beta}{c}\right) \frac{1-\exp\left(-2\beta\rho\right) }{\rho}  $$

(Exactly the same results may be obtained by using the usual three-dimensional Green function 1/*r* and the Bessel expansion thereof but the derivation then is more, and unnecessarily, complicated). Again, by introducing the harmonic potential *A* ln*ρ* to cancel the streaming at *ρ*=*ε*, we obtain in exact analogy to the spherical case that the streaming velocity is independent of the hydraulic conductivity and of the streaming force and is given by

65 $$ \gamma\mathbf{v}=:\gamma v_{C}\hat{\mathbf{\rho}}  $$

where

66 $$ v_{C}=\frac{P}{2\pi}\frac{\phi_{0}}{\rho_{0}c^{2}}\times\frac{\exp\left(-2\beta\varepsilon\right) -\exp\left(-2\beta\rho\right) }{\rho}   $$

for *ρ*>*ε*.

#### The circular piston radiator

In the above, we added a harmonic potential to cancel the streaming at the acoustic source arising from both the Green function and the source arising from Reyold’s stress. In the case of a planar piston radiator in a baffle, as well as the next one we treat below, we are concerned with streaming within a half space, and so it is just as easy to use the reflecting Green function

67 $$ G_{N}\left(\mathbf{x},\mathbf{x}^{\prime}\right) :=\frac{1}{\left\vert \mathbf{x-x}^{\prime}\right\vert }+\frac{1}{\left\vert \mathbf{x+x}^{\prime }\right\vert }  $$

so that there is no contribution at the plane (considered to be at *z*=0). Then we need the harmonic potential to cancel only the streaming arising from **f**. The acoustic field from the circular piston radiator, is remarkably rich. We find that the formulas from [29] are among the most complete and useful for general purposes, although their purpose was quite different and meant to expose the fascinating pattern of wavefront dislocations in such a radiation field. Many references on acoustics discuss the fields from such a radiator, but are usually satisfied with giving approximate expressions under various conditions, e.g., on-axis, in the far field, and so on. We shall not violate this tradition and use the following crude approximation to compute the radiation fields and the resulting streaming. In this case we shall compute the streaming only on-axis, and we make the following simple approximations. We use the notation introduced in (43b) to write

68 $$ p_{2}=\frac{1}{4\pi\mathcal{D}}q\ast G_{N}  $$

**Fig. 1:**
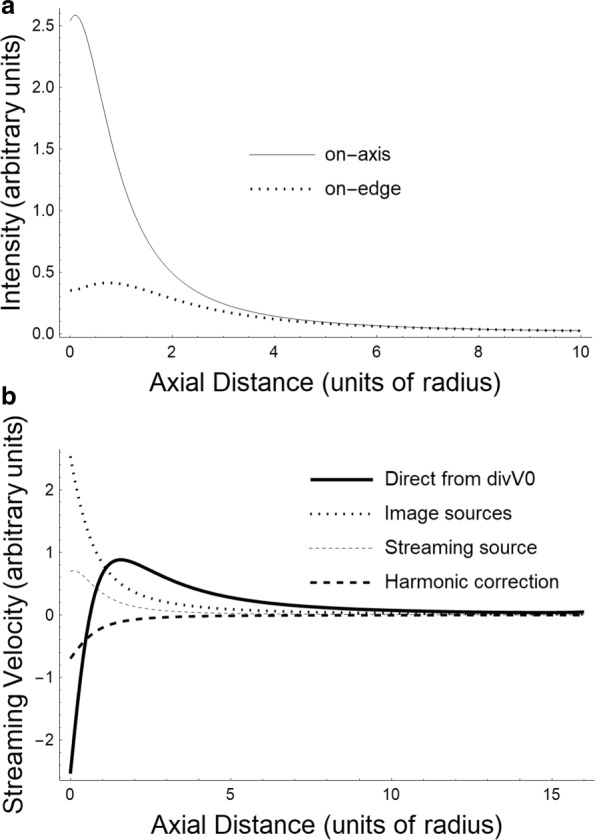
The piston radiator. **a** On-axis and on-edge intensities. **b** Contributions to the streaming velocities in the calculational method. As the text indicates, we use the average of the on-axis and on-edge intensities displayed in (**a**). Our calculation uses the reflecting Green function which is a sum of two terms shown in (**b**) as “direct” and “image” sources. The contribution of the streaming force and finally that of the harmonic function needed to cancel out streaming on the plane of the radiator are also shown. The total streaming velocity is a sum of all these and is dominated by the direct Green function a little bit away from the radiator

We shall use the *dimensionless*cylindrical coordinates (*z*,*ρ*,*ϕ*) introduced in [29]. Further, we shall use a drastic approximation for the intensity (and thus for *q* as well). We take the expressions given in [29], and simply average the on-axis and the on-edge intensities (displayed in Fig. 1a), so that the expression for *q* is of the form

69 $$ q=h\left(z\right) \Theta\left(1-\rho\right)  $$

where *h*(*z*) is the average just mentioned, and *Θ* is the Heaviside unit function. Thus *q* is constant in the radial direction, and zero outside the circle bounding the radiator. Further using the expansion of the Coulomb kernel in terms of the modified Bessel functions *I*_*m*_,*K*_*m*_, [22], we eventually find

70 $$ {\begin{aligned} q\ast G_{N}&=2\pi\int_{0}^{\infty}dz^{\prime}q\left(z^{\prime}\right) \left[ \sqrt{1+\left(z-z^{\prime}\right)^{2}}-\left\vert z-z^{\prime }\right\vert\right. \\ & \quad +\sqrt{1+\left(z+z^{\prime}\right)^{2}}-\left\vert z+z^{\prime}\right\vert\left. {\vphantom{\sqrt{1+\left(z-z^{\prime}\right)^{2}}}}\right] \end{aligned}}  $$

To find the potential required to cancel the streaming from the source term, we write a harmonic potential

71 $$ \psi\left(z,\rho\right) :=\int_{0}^{\infty}dk\text{ka}\left(k\right) \exp\left(-kz\right) J_{0}\left(k\rho\right)  $$

and determine the coefficients *a*(*k*) by matching at the boundary. We find

72 $$ {\begin{aligned} \psi\left(z,\rho\right) \!:=-\!\frac{2\beta}{c}h\left(0\right) \int_{0}^{\infty}dk\text{ }\frac{J_{1}\left(k\right) }{k}\exp\left(-kz\right) J_{0}\left(k\rho\right) \end{aligned}}  $$

The streaming speed on-axis is in the *z*-direction and is

73 $$ \gamma v=-\frac{1}{4\pi\mathcal{D}}\nabla\left(q\ast G_{N}\right) -\nabla\psi+\frac{1}{c}q  $$

This expression is evaluated numerically and will be used in the next section. However, the terms arising from our decomposition, evaluated for a particular geometry (the diameter or aperture of the circular piston was in this case 0.64 cm), are shown in Fig. 1b to illustrate the cancellations at the origin to give zero streaming at the boundary. From the figure, it is also apparent that, beyond a distance of about the width of the aperture, the conservation force alone determines the streaming velocity. We should emphasize that this distance in fact is far less than the far field distance which was 14 cm. This result, namely *the ineffectiveness of all the terms but the pressure gradient arising from the infinite space Green function, is true for all the cases we have examined*. In particular, the streaming force is not significant, as our order of magnitude argument in the “Streaming in porous media” subsection indicated. This also means that the hydraulic conductivity of the medium is irrelevant.

#### Focused spherical transducer

We use the approach described above for the piston radiator, along with the intensity patterns appropriate for the focused spherical transducer which are given for example by Kino [26] for details. However, we shall simplify the expressions still further, using a Gaussian paraxial approximation to the beam profile. We account for attenuation by the approximation

74 $$ \left\langle \mathbf{I}\right\rangle \left(\rho,z\right) =\left\langle \bar{\mathbf{I}}\right\rangle \left(\rho,z\right) \exp\left(-2\beta z\right)  $$

where we force $\bar {\mathbf {I}}\left (\rho,z\right) $ to be divergence free. Then,

75 $$ \nabla\cdot\left\langle \mathbf{I}\right\rangle =-2\beta\bar{I}_{z}\left(\rho,z\right)  $$

The solution for the pressure using the reflecting Green function becomes

76 $$ {}p=\frac{2\beta}{4\pi\mathcal{D}}\int_{0}^{\infty}dz^{\prime}\int_{0}^{\infty }d\rho^{\prime}\rho^{\prime}\int_{0}^{2\pi}d\phi^{\prime}\bar{I}_{z}\left(\rho^{\prime},z^{\prime}\right) G_{N}\left(\mathbf{x},\mathbf{x}^{\prime }\right)  $$

Here we have used the same Green function as for the piston radiator, in effect turning the spherical cap that is the radiator into the disk that is the projection of the radiator onto its base plane. In fact, the solution to the Neumann problem for a spherical cap is readily available, see [30]. However, we content ourselves with the approximation which results in much simpler formulas. Again, the justification is to first take a crude look at the results before investing in what could turn out to be pointless refinements. This pressure has no normal gradient on the plane of the radiator and the baffle. Our approximation is that the *unattenuated*(period-averaged) intensity in the *z*-direction is given by

77 $$ \bar{I}_{z}\left(\rho,z\right) =:\frac{P}{2\pi\sigma^{2}\left(z\right) }\exp\left(-\rho^{2}/2\sigma^{2}\left(z\right) \right)  $$

where *P* is the power emitted into the porous medium. With proper choice of *σ*(*z*), this will agree approximately with the profile given by Kino along the *z*-axis, and also ensure that the integral over any constant −*z* plane reduces to the total power,

78 $$ 2\pi\int_{0}^{\infty}\frac{P}{2\pi\sigma^{2}\left(z\right) }\exp\left(-\rho^{2}/2\sigma^{2}\left(z\right) \right) \rho d\rho=P  $$

We choose

79 $$ \sigma^{2}\left(z\right) =:\sigma_{f}^{2}\left(1+G^{2}\left(z-f\right)^{2}\right)  $$

We obtain *G* from a least squares fit to the axial intensity profile (*ρ*=0) and *σ*_*f*_ from that to a radial intensity profile, using Kino’s expressions (see Eqs. (3.3.13) and (3.3.24) *et seq*in [26]). For numerical evaluation below, we have chosen the geometric parameters to be those for a focused transducer made by FUS Instruments Inc., Toronto, Canada, and used in an experiment, with which we shall compare below. This transducer has a focal length *f*=4 cm, and the half-aperture of the spherical transducer *a*=1.25 cm. We obtained

80 $$ G\approx7.84\text{ }\operatorname*{cm}{}^{-1};\text{ }\sigma_{f} \approx0.02\operatorname*{cm}  $$

**Fig. 2:**
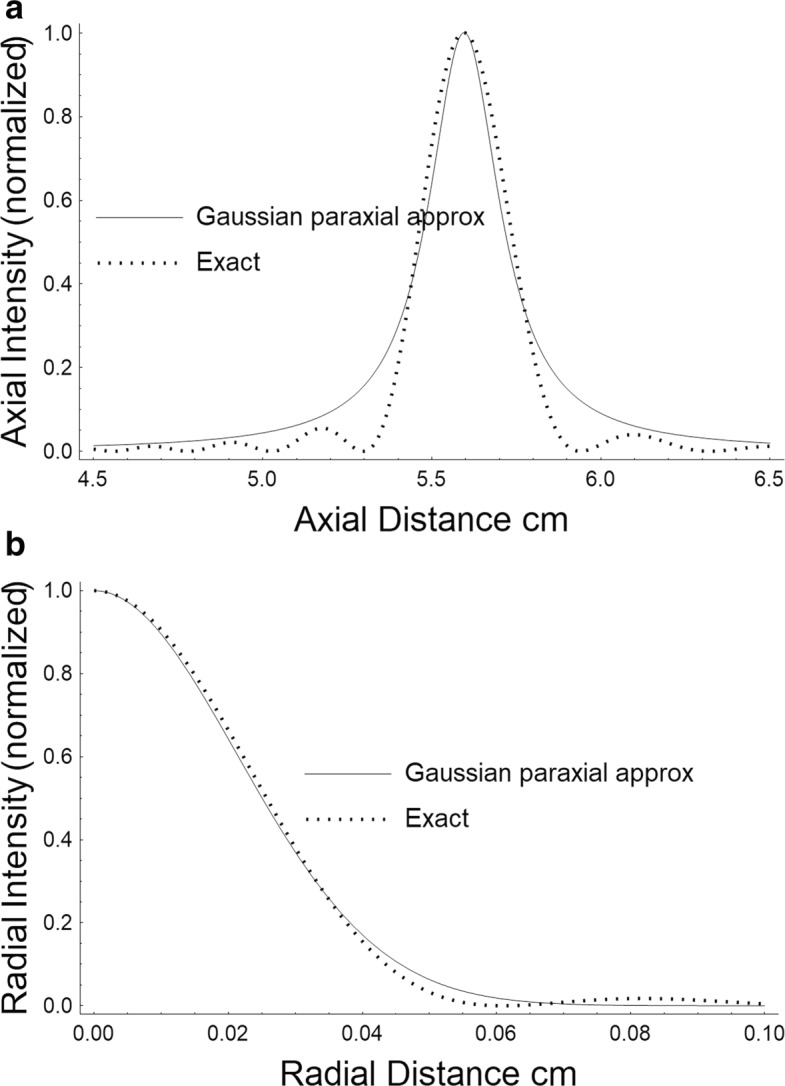
**a** Comparison of axial intensity patterns. **b** Comparison of radial patterns. A comparison of the paraxial Gaussian beam intensity patterns of a focused radiator with more exact diffractive patterns. The on-axis intensity versus axial distance and the off-axis intensity at the focus versus radial distance are displayed in the two figures

In Fig. 2, we compare the relative on-axis intensities due to our paraxial Gaussian approximation with one taking into account the interference of the waves as given by Kino [26]. The intensity units are arbitrary and the figure is meant to illustrate only the relative magnitudes of the two curves. Figure 2a is for the axial and Fig. 2b for the radial profile and in each case a normalized profile is displayed. We can see that our approximations are quite close to the exact results. We use cylindrical coordinates so that

81 $$ {\begin{aligned} \left\vert \mathbf{x}\,-\,\mathbf{x}^{\prime}\right\vert \,=\, \sqrt{\left(\rho^{\prime}-\rho\right)^{2}+\left(z^{\prime}-z\right)^{2}-2\rho \rho^{\prime}\cos\left(\phi^{\prime}-\phi\right) } \end{aligned}}  $$

Further, we evaluate only on-axis (*ρ*=0). We get

82 $$ {{}\begin{aligned} p\left(\rho=0,z\right) & =\frac{P\beta\sqrt{\pi}}{4\pi\mathcal{D}} \int_{0}^{\infty}dz^{\prime}\frac{\exp\left(-2\beta z^{\prime}\right) }{\sigma\left(z^{\prime}\right) \sqrt{2}} \times\left\{ \exp\left[ \frac{\left(z^{\prime}-z\right)^{2}}{2\sigma^{2}\left(z^{\prime}\right) }\right] \operatorname{erfc}\left[ \frac{\left\vert z^{\prime}-z\right\vert }{\sigma\left(z^{\prime}\right) \sqrt{2}}\right] \right.\\ &\left. \quad +\exp\left[ \frac{\left(z^{\prime}+z\right)^{2} }{2\sigma^{2}\left(z^{\prime}\right) }\right] \operatorname{erfc}\left[ \frac{\left\vert z^{\prime}+z\right\vert }{\sigma\left(z^{\prime}\right) \sqrt{2}}\right] \right\}  \end{aligned}}  $$

The gradient in the *z*-direction is easily obtained by noting

83 $$ \frac{d}{dz}\operatorname{erfc}\left[ \left\vert z\right\vert \right] =-\frac{2}{\sqrt{\pi}}\exp\left(-z^{2}\right) \operatorname*{Sign}\left(z\right)   $$

On the other hand the streaming force is

84 $$ \mathbf{f}\left(\rho=0,z\right) =\frac{2\beta}{c}\frac{P}{2\pi\sigma^{2}\left(z\right) }\exp\left(-2\beta z\right) \hat{\mathbf{z}}   $$

so that on the axis and on the radiator/baffle it is

85 $$ \mathbf{f}\left(\rho=0,z=0\right) =\frac{2\beta}{c}\frac{P}{2\pi\sigma_{0}^{2}}\exp\left(-\rho^{2}/2\sigma_{0}^{2}\right) \hat{\mathbf{z}}   $$

where

86 $$ \sigma_{0}^{2}:=\sigma^{2}\left(0\right) =\sigma_{f}^{2}\left(1+G^{2}f^{2}\right)  $$

The harmonic potential *ψ* required to cancel the effect of the streaming force (85) on the radiator is of the same form as for the piston, though with a different constant of course. Its gradient turns out to be

87 $$ {}-\frac{\partial\psi}{\partial z}\left(\rho\,=\,0,z\right) \,=\,\frac{\beta P}{\pi c}\!\left\{\! \frac{1}{\sigma_{0}^{2}}\,-\,\exp\!\left(\! -\frac{z^{2}}{2\sigma_{0}^{2} }\!\right)\! \sqrt{\frac{\pi}{2}}\operatorname{erfc}\!\left[\! \frac{z}{\sigma_{0}\sqrt{2}}\right] \right\}  $$

With this, the streaming velocity along the axis evaluated exactly on the axis is given by

88 $$ v_{F}\left(z\right) :=K\left(-\frac{\partial p}{\partial z} -\frac{\partial\psi}{\partial z}+\mathbf{f}\right)   $$

This equation is evaluated using (83) on (82) to obtain the pressure gradient, and (84) to obtain the streaming force. The numerical results are described in the next section.

## Ultrasound-enhanced convective delivery (UeCD)

**Fig. 3:**
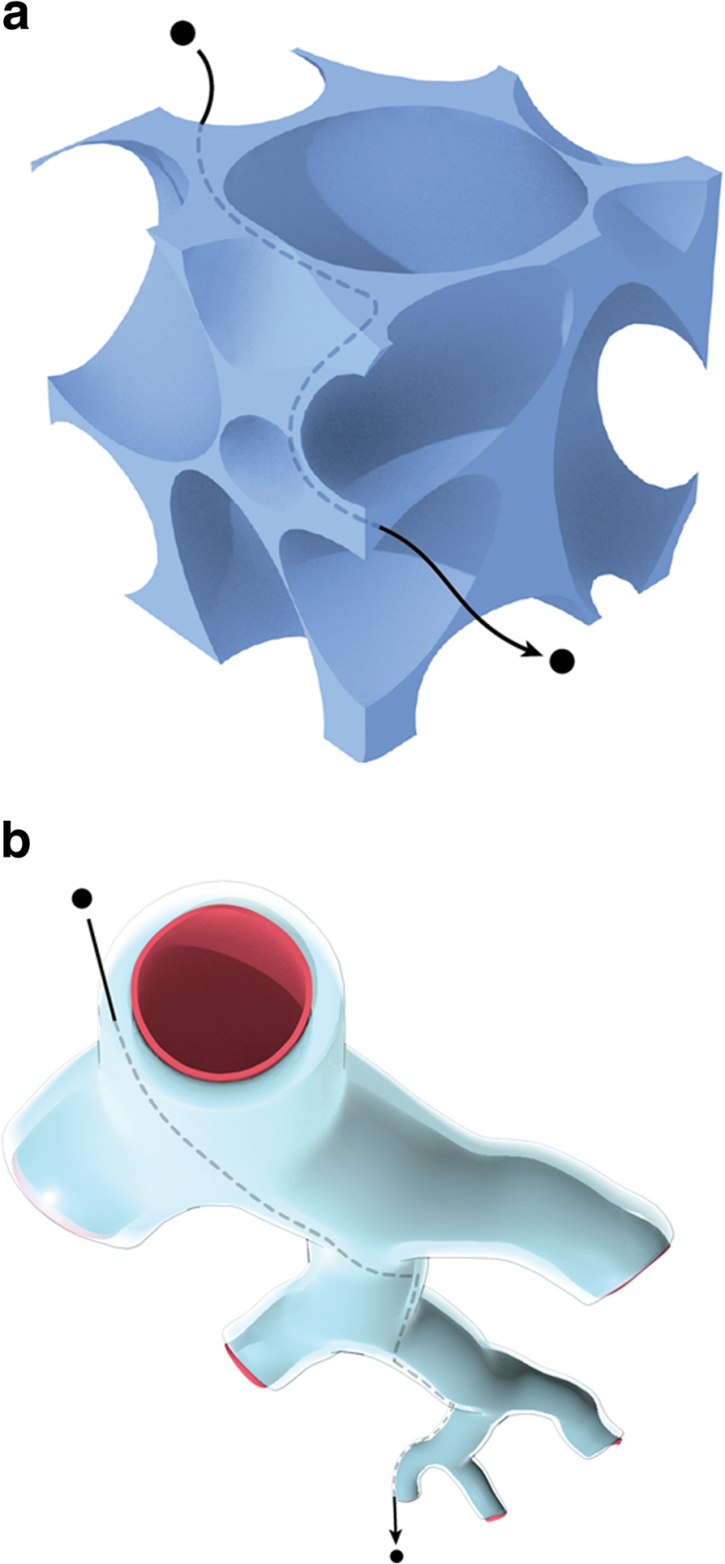
Pathways for particle transport within brain parenchyma. **a** An illustration of the conventional interstitial pathway available to a molecule or particle. **b** The perivascular pathway for particles: larger particles may prefer such pathways to the interstitial

One impetus for the theory developed above has been to examine if streaming can play a role in enhancing the spread (i.e., increasing the range) of therapeutic particles injected into the tissue. Such techniques are under experimentation in animals, from rodents to monkeys, and we compare the results of calculations of streaming with those of the experiments in live brains (along with one *ex-vivo*brain experiment). It is of some interest perhaps to illustrate two of the pathways available to such particles within the brain. One is the traditional interstitial space (of volume fraction we have denoted by *ϕ*) shown in blue in Fig. 3a outside the cells that are shown as empty white spaces. As stated in the Introduction, the widths of these spaces between cells can be taken to be of the order of 100 nm. If a particle cloud fills these spaces up to a volume *V*_*d*_ (which is called the volume of distribution), the actual volume of the fluid in which these particles are suspended (called the infusion volume) is obviously only *V*_*i*_:=*ϕV*_*d*_. Of course, in practice, one is given *V*_*i*_, and *V*_*d*_ is measured in some way (subsection immediately below) to allow one to infer how far the drugs or marker particles have traveled. These interstitial spaces have been known for a long time [17], but more recently, people have discovered the hidden potential of another pathway illustrated in Fig. 3b: see e.g., [31] and also Appendix 1 of the opinion article [32] referring to the potential of these spaces in understanding drug delivery. These perivascular spaces are narrow annular spaces (but with widths of the same order of magnitude as of the interstitium!) of high fluid conductivity surrounding arteries. They were discovered in the 19^*th*^ century (and have been also known as the Virchow-Robin spaces), but were surmised to be restricted to major arteries and end at shallow depths into the brain after the entry of the blood vessels in brain parenchyma. In this century however, they have been shown to extend deep into the brain, down to the level of small blood vessels and so may serve to spread a particle quite far from its entry point into a perivascular region, particularly because these pathways are more conductive than the volumetric interstitial spaces. Effectively, given the density of the blood vessels, this makes *ϕ* very small (*V*_*d*_ very large for a given *V*_*i*_) but it should be noted that the distribution will be more sparse than for the conventional interstitial spaces which surround every cell, in contrast to the perivascular spaces surrounding blood vessels which are of course more sparse than cells. However since it is estimated that there is a capillary for every few cells, diffusion for small distances from the perivascular space into the surrounding tissue may be effective in delivering free drugs to the target regions. This diffusion may also be aided by sonication, though of course our theory does not cover this phenomenon.

### Measures of enhancement

The standard measure of how a molecule fills tissue is the volume of distribution *V*_*d*_. This term is used quite differently in the field of intraparenchymal delivery than in pharmacokinetics (though the intent is the same) and it is therefore important to understand how it is ubiquitously (with rare exceptions) used and measured. It is most often used in-vivo, particularly with magnetic resonance imaging (MRI) contrast reagents such as gadolinium (chelated), and with the exception of our own work (see, e.g., [4] for a variety of sizes of such contrast reagents), *V*_*d*_ is measured by threshold of the intensity of the MR (image) – see the experiments quoted below. Such a distribution volume has very little quantitative significance because (i) it is highly dependent on the threshold; and (ii) the image intensity has no linear relationship with concentration of the contrast reagent in MRI (though it does in X-ray computed tomography (CT)). However, it is certainly true that if the same method is used with and without sonication, the relative *V*_*d*_’s are an indication of the effectiveness of the sonication in increasing the spread. Due to the pronounced irregularity of the distribution in tissue and the reasons just mentioned, we have chosen rather the distance or range of a particle to compute and to compare with experiment. This would also generalize to more restricted pathways such as the perivascular where the concept of *V*_*d*_ would not be useful. The method by which we obtain this from the experiments is briefly described below; that from the theory has already stated in Eq. (44). The enhancement due to the sonication in UeCD is measured experimentally by the extra range of the particle over the duration of an experiment. For theoretical purposes, since it is the time that is directly computed by (44), we have compared the duration of the experiment versus what the theory would predict would be required to obtain the required enhancement of range. It is a trivial numerical matter to fix the time and compare the ranges but we have chosen not to do so.

### Comparisons with experiments

Table 1 summarizes important parameters for the experiments we have examined. The column entries are as follows: the first gives the reference for the experiment, the second gives the strength of the acoustic source: either a reported pressure in kilopascals or a reported intensity converted to pressure using the plane wave approximation. The third column reports where, with respect to the position of the sound source along the axis of the CED catheter, the source strength was measured. The fourth column reports a radial or axial distance of enhancement of transport of the solute (with sonication as compared to without) as inferred from the experiments. The final column gives the effective time of sonication in minutes. Many of the experiments involve a pulsed source and the overall time was multiplied by the duty cycle fraction to obtain this number. The form of the source was also very variable: these, as well as the operating frequencies, are mentioned in the discussion below under a listing each of the experiments. The last two rows refer to as yet unpublished results: see under these experiment listings below for further details.

**Table 1:** Important parameters for the experiments examined for comparison with theory

Experiment	Source strength (KPa)	Where measured: mm from source	Distance enhanced − mm (measured)	Sonication time (min)
[33]	125	0	1	2
[34]	1240	4.2	3	4.5
[35]	30	5.5	0.13	30
[36]	150	5.5	0.35	30
[37]	3	2 (from rod tip)	1.37	140
Cornell-Weill(2017)*	1700	56 (focus)	1.3	2
Therataxis (2017)*	0.303 W/cm	0	2.5	105

The starred experiments are yet to be published

Before making some numerical estimates, we point out that in all the experiments considered, the amount of fluid available for streaming is fixed entirely independent of the acoustics. As an example, consider experiments in which sonication has been used along with fluid delivery into brain tissue through a catheter. In these, the fluid was delivered through a pump that introduced a fixed volume of fluid per unit time. All of the transducers used in the experiments examined have an axis of symmetry, and the theory is then pertinent for the speeds along this axis, less so off-axis, due to the circulation that is demanded by mass conservation. Also, we have completely ignored the temperature rise that would be consequent upon sonication, and its effects on the tissue. It is not hard to write down equations for the temperature distribution, but the scope of this paper is only to indicate the simplest (but “no simpler”!: see the extended discussion in the “Streaming in porous media” subsection) equations for streaming in a porous medium. We also ignore the impact of the acoustic waves on the medium properties. We have for example assumed the hydraulic conductivity to be fixed. However, if the hydraulic conductivity change can be separately estimated, we can certainly include it in the calculation. It turns out that the results are essentially independent of the hydraulic conductivity for the cases we have examined (see remarks at the end of the “The circular piston radiator” subsection above). The theory does not compute streaming the presence of the background flow due to the convective delivery from the catheter that is concomitant with the ultrasound in most of the experiments. *In other words, we examine the effects of CED and of the ultrasound as if they were additive*. Some further parameters we have used are: the hydraulic conductivity *K* is assumed to be 2×10^−7^ in cgs (cm^4^/ dyne− sec) which is close to the value quoted in a seminal paper on CED from the NIH group that originated it [25] (though as just stated this makes no difference); the acoustic attenuation coefficient *β* is taken to be 0.16 cm^−1^ at 1.5 MHz [9]; the speed of sound in tissue is 1.6×10^5^ cm/ sec; and the viscosity of water to be that at 37 C, 0.007 dyne− sec/ cm^2^.

Table 2 summarizes our findings for in-vivo experiments and some further remarks are made within the listing of the experiments below. A condition that pertains to almost all of the experiments is that the observed transport took place well before the far field distance of the source was reached (i.e., within the near field). We do not provide details of the published experiments: the sources cited may be consulted for these. All the programs were constructed in *Mathematica*11 and implemented the theory described, using the particular parameters from each experiment in addition to the other tissue parameters mentioned just above.

- ***Ex-vivo**** infusion in brain tissue sample* [33]; 1.58 MHz**focused spherical radiator.** Fom Fig. 3 of their paper, it would seem that the distance penetrated in equine brain tissue is close to 1 mm, and our estimated effective sonication time agrees with the experiment, well within the uncertainties of the measurements themselves.

**Table 2:** A comparison of theory with experiment for the particle range enhancement due to sonication

Experiment	Distance enhanced − mm (meas)	Experimental sonication time (min)	Theoretical sonication time (min)
[33]	1	2	2.5
[34]	3	4.5	0.5
[35]	0.14	30	35
[36]	0.35	30	28
[37]	1.37	140	24 days?? see text
Cornell-Weill(2017)*	1.3	2	6
Therataxis (2017)*	2.5	105	800

See also Table 1 for meaning of the asterisks
- **In-vivo infusion in primate with intermittent sonication** [34]; 1 MHz**piston radiator.** This experiment was the first one (as far as we are aware) to use ultrasonic enhancement of CED drug delivery in-vivo. This and the following three experiments are in-vivo with an intact brain within skull. Sound waves within a brain enclosed by a skull will be subject to reflections, standing waves, as well as mode conversions – a situation quite different from our calculations for progressive longitudinal waves. Nevertheless, we can investigate some order of magnitude effects of streaming theory and compare with the results. All the results that are germane to our theory for the present case are summarized in Table 1 of [34]. Their paper is somewhat confusing but referring to a trial which showed no enhancement of distribution volume (!), they report that “distribution...was enhanced from 9 mm to 12 mm due to the sonication”. Assuming this to be true, (given their source strength one would indeed expect some effect), theory and experiment are well within the same order of magnitude. However, this seeming agreement could be entirely coincidental since the interpretation of their results is very problematic.
- **In-vivo infusion in rodent** [35]; 1.34 MHz**piston radiator.** In this and the next reference, there were a subset of experiments in which microbubbles were used: we shall not consider those experiments since their interpretation is beyond the scope of this paper. Only the experiments comparing CED with and without sonication but with no microbubbles are compared here. The distributions were measured by areas of stained sections. The top left graphs in Figs. 7 and 8 of that paper show the areas of coronal sections at various distances along an axis perpendicular to these sections. The area of the central section within Fig. 7 is about 0.75 mm^2^. If we take this to fill a disk (see the idealized illustration in their Fig. 2, right), then the radius of the disk is about 0.49 mm. Similarly the bottom figure shows a central section after sonication with an area of about 1.25 mm^2^, corresponding to a radius of 0.63 mm. The increase in radial distance is about 140 microns. By using the measurements quoted in Table 2 to obtain the power of the equivalent spherical source, we obtain that increase of radial distance in under 35 min of sonication, as compared with the half hour reported. This level of agreement is really suspiciously good given the crudity of both the theoretical approximation and the experimental protocol to obtain the areas.
- **In-vivo infusion in rodent with time-reversal acoustics** [36]. In this experiment, the results from their Fig. 5 for Evans blue dye indicate that the extra radial distance due to sonication is about 350 microns. This is obtained exactly as before by computing radii of a disk with the areas shown for the central slice. We should also point out that this experiment used time reversal acoustics with a reflector on the skull, thus reinforcing the presence of standing waves, so that the theory above is not directly applicable. However, again in the spirit of seeing if there is any rough agreement, we may use the same results we developed for focused spherical radiator. We cannot, given the data available, form a good estimate of the intensity patterns due to the time reversal focusing with their reverbator. So again, the agreement between experiment and theory in Table 2 must be regarded as fortuitous.
- **In-vivo infusion into primate** [37]; 300 kHz**acoustic horn.** This experiment used an acoustic horn to focus the sound onto a steel rod which was introduced into the tissue. The steel rod contains the catheter lumen as well, and the pressure from the sonication was measured in water just below (2 mm) the catheter lumen and steel rod. We made no attempt to compute the acoustic fields resulting from this configuration, but considered simply a spherical radiator source at the rod tip, with pressure that was measured to calibrate the source power. Further, the radial distances which we compare (under CED conditions, and with and without sonication) are obtained by using their volumes of distribution (Fig. 8C of the cited reference). We estimate the radius of that volume assuming a spherical distribution (which it manifestly is not as can be seen from their Fig. 8A). It would have been somewhat more reliable to use a section which might show a disk (circle) for the distribution, but that data was not available. In this case, as can be seen from Table 2, our luck has seemingly completely run out. While our assumptions on the form of the source may have introduced some error, we are rescued by another consideration in this case. The electronics and the radiation pressure changes involved in the pulsed excitations induces a strong lateral force that can deflect the steel rod. This is easily observable in fluids with soft catheters. In a conversation with the senior author of this investigation, it was confirmed that strong deflections of the rod were indeed observed. Thus we surmise that the enhancement – which, it must be remembered, is still very small by any macroscopic considerations – has nothing to do with the effects of acoustic radiation within tissue, but rather with the vibratory deflections of the steel rod due to the much lower frequency of the on-off pulsed transmission that was used. We will therefore ascribe these published results as irrelevant to Acoustic Shepherding or to UeCD as understood here.
- **In-vivo infusion with focused ultrasound (unpublished)**; 1 MHz**focused spherical radiator.** A focused radiator made by FUS Instruments^2^ was used in experiments reported at a conference. The publication has not appeared at the time of this writing. The authors report directly an increase from 4.2 mm to 5.5 mm of the range of tracked MR contrast reagent particles (Gadolinium). In this case, our theoretical sonication time seems a little too long. However, again, we have a mitigating circumstance. The focused intensity is relatively large (and this is the intensity that applies at the point of transport). It has been well known that focused beams considerably increase the attenuation due to nonlinear effects [38] and while we have not investigated the modified form of the Gaussian beam with nonlinearities, a large increase in pressure gradient due to such nonlinearities seems well within possibility as shown in [39]. This would serve to correspondingly decrease the time and we regard that our results are also consonant with this experiment as well.
- ***Ex-vivo****infusion in porcine liver (unpublished)*; 1.68 MHz**radial cylindrical pulsation.** We have undertaken studies using an EkoSonic Mach4™sonicated catheter along with control unit. The device has a linear array of several transducers in an inner lumen surrounded by an annulus through which fluid flows out of a multitude of microports into the tissue. We approximate this as a cylindrical sonicator with radial pulsation treated in the “Cylinder with radial pulsations” subsection^3^. We computed the radial enhancement of particle distance as follows. The experiments were conducted under X-ray CT imaging, using Visipaque™contrast reagent. (We were thus able to measure the concentrations of the marker particles accurately, in contrast [no pun intended] to the other experiments reported here). In any case, we conducted three pairs of experiments where the proximal/distal half of the length of the catheter was sonicated allowing us to compare the radial distances to which the contrast traveled. We measured such distances in the centers of the sonicated/unsonicated region and obtained about ${\frac 12}$ cm or more of enhanced transport over a three hour infusion. However, it turns out that the sonicator was degraded considerably during the second half of the experiment. We took the best experiment (in terms of distance enhancement) during the first half of the experiment, and compared with the theory. The theoretical time indicates an efficiency about 8 times worse than the best performing experiment. Again, given the uncertainties and heterogeneity of tissue, and that we chose the maximum distance attained in the experiments, we regard this also as within an order of magnitude agreement, though only just.

## Conclusions

The theory of streaming, compared with experimental results, seems to suggest that such streaming in tissue may be of similar order of magnitude as regular infusions that are undertaken to spread drug into brain and other parenchyma, in contrast to widespread belief that it should be entirely ineffective. This theory also agrees surprisingly well with experiment. However, there is at least one important feature in all of the experiments available which seriously limits the usefulness of such applications of UeCD. Namely, the distances of enhancement have been generally sub millimeter, and the total distances of transport of the order of a few millimeters. One point to make is that such transport away from the catheter can easily be achieved by CED alone by simply increasing the flow rate: one hardly needs the assistance of sonication to reach millimetric distances. Of course, the counter would be that these are very early times for these studies. While true, limiting the distances to such small values also means that we cannot separate confounding effects such as simply the mechanical vibrations transmitted through the elastic framework having some effects that would not be accounted for by the sound waves through (mostly) the fluid. Thus both for fundamental and practical reasons, it seems important to overcome the limitations of the current generation of experiments, without compromising the safety of the sonication in living biological tissue. Another point following upon the above and which deserves emphasis is that it is indeed relatively useless to confine the application of sonication pressure to the same place as that of the pressure driving fluid flow. (This point was already made slightly differently in the Introduction). There was only one exception to this, namely the yet unpublished results from the Cornell Medical Center (New York). Other than this study, the catheter port even in [36], which used the time reversal method to focus the sound, was chosen as the focal poin, reducing the potential impact of UeCD. After all, the selling point of this method is that we focus the sound intensities to provide pressure gradients anywhere as opposed to the catheter which provides the pressure only at the port or ports of the catheter. Thus the true benefit of UeCD remain to be explored.

## Acoustic effects in structured porous media

In the above, we have examined porous medium equations under what we have called the *Imaging Community*assumption (see “Introduction”) where the ultrastructure of the porous medium is not visible to the acoustic wave propagation. We examined various phenomena under this assumption and came up with the basic equations for acoustic streaming. In comparison with studies in tissue, it seems that the streaming discussed so far seems adequate to explain the results. However, in this section, we point out some further phenomena that reveal themselves when we do consider the ultrastructure, and indicate why they are unlikely for the most part to play a role in UeCD. Before we do, we indicate why we have largely ignored the vast field of biological effects of sonication. Throughout, our considerations are confined to the cases with no microbubbles, resonant or otherwise.

### Biological effects of sonication

There is an enormous literature on biological effects of ultrasound: we merely point to [40–42] as a few examples of many discussions, none of which are relevant to volume transmission of fluid. We have pointed out that an effect generally been considered to be non-existent except in fluid-filled spaces, may however be visible in the creeping flows currently used in clinical trials of intraparenchymally induced drugs [43, 44]. However, the way in which bioeffects of ultrasound can affect our treatment is by suggesting different assumptions. As stated, we do not consider exchange of water between intra– and extra–cellular compartments. We cannot assess if this would contribute in any way to macrostreaming. Another effect, argued for in [45] is that an acoustic wave does alter the interstitial volume fraction *ϕ*, and thereby the fluid conductivity *K*. However, see our discussion of this point in the “Comparisons with experiments” subsection. The purpose of these authors was entirely different: increasing *ϕ* allows larger particles that would otherwise not be able to traverse the interstitial spaces to do so. We have always assumed free advection of particles in the fluid, and so this effect is not germane to the present treatment. We have also not treated the diffusivity of particles in our equations, assuming that all the effects we see (particularly for short times) would be due to advection. There is a well known effect of enhancement of diffusivity due to wave action, which were investigated long ago [46], and continued to be more recently [47]^4^. A rigorous treatment of the mathematics of this is given in [48] but for the acoustic intensities we have considered, and the small diffusivity of the tracers, this effect is even more negligible than the streaming we have calculated (*Application* *3* of the cited reference is the most relevant for our consideration). So, we now turn our attention to phenomena we have not treated, but which depend on the fact that that medium has a microstructure.

### Acoustic microstreaming

In this section, we argue that the widely examined circulatory microstreaming, due to the presence of solid boundaries such as the cell walls, is unlikely to enhance the transport of fluid or solutes carried by it. Such streaming – not considered in our theoretical development above – has received most of the attention in the literature, though not in the context of a dense distribution of cells. Nyborg, a pioneer in acoustic streaming as indicated above, has also been a pioneer in investigating the biological effects of ultrasound (see the reviews [41, 49, 50], which all contain references to earlier work and primary sources). We would expect the direct effect of circulatory streaming to be null: the velocity field circulates. However one might speculate that “hydrodynamic dispersion” might enhance the transport. This mechanism has been well known to hydrologists for a long time (see the chapter of that name in [51], where references to the original literature where these concepts were introduced – [52, 53] and others – are given). The type of hydrodynamic dispersion that could play a role is what Bear calls “mechanical” dispersion though that name has gotten out of favor: it is due to the mixing of the particles traveling down different channels so that even though the Darcy velocity field has a definite direction, the microchannels along which the particles move causes a dispersion which mimics a diffusivity that depends on the magnitude *V* of the Darcy velocity (see the reference for a detailed explanation and the conditions under which a mean square deviation is equivalent to a diffusivity, which demands a Gaussian distribution). There is another form of dispersion, more celebrated, called Taylor or Taylor–Aris dispersion which involves mixing within a channel. We ignore this because the channels are quite narrow and short. Now, if we create a random network of channels with characteristic length *L*, then over long times, we may expect a diffusivity ∼*LV* from dimensional analysis. The microstreaming velocity which occurs within the boundary layer depends on the displacement amplitude *A* of the cell walls, the frequency *ω*, and the characteristic dimension of the cell. Estimates suggest

89 $$ V\sim\omega A^{2}/L   $$

which would result in an effective diffusivity

90 $$ D_{\operatorname*{eff}}\sim\omega A^{2}   $$

To estimate the displacement amplitude, we use the particle velocity of the homogenized medium as discussed above, and determine the displacement over a half period from that. In even the small intensities used in the Cornell in-vivo experiments, this results in *V*∼0.1 mm/ sec at the cannula tip which results in dispersive diffusivity *D*_*H*_∼2×10^3^ *μ* m^2^/ sec. Such a high dispersivity would result in a root mean square dispersion $r_{H}:=\sqrt {4D_{H}t}$, under spherically isotropic conditions, for the particle. In 20 minutes, this is of the order of a few millimeters, much larger than the spread observed in the experiments described. In the classical treatments, the diameter of the microstreaming paths is of the order of a wavelength (1 mm in this case). This is indeed much larger than *L* so we may expect brancing into various channels and enhancement of dispersion of solutes.

However, the flaw with this argument is that it fails to take into account the packed cells in tissue, which will likely nullify any microstreaming effects. The above estimate was based on an isolated cell. However, from the extensive work conducted in the 1950’s on such solutions for a boundary in the fluid – see for example [54, 55] – it may be seen that the microstreaming velocity just estimated does not reach that value till a distance from the boundary a few times the cell radius, being a very small fraction of it within a cell radius. As we have pointed out, the interstitial widths is about two orders of magnitude smaller than a cell radius. Thus intuition would suggest that the intercellular distances being all well within the width of a boundary layer, and there is no room for a streaming effect to sustain itself. We can develop this a bit further, leaving a more detailed verification to future numerical simulations. In a fluid with an internal boundary, and restoring subscripts denoting the order of the quantities, recall that the second order perturbation equations for the streaming are:

91 $$\begin{array}{*{20}l} -\eta\Delta\mathbf{v}_{2}\mathbf{-}\nabla p_{2} & =\mathbf{-}\nabla p_{2}+\mathbf{f} \end{array} $$

92 $$\begin{array}{*{20}l} \mathbf{f} & :=-\nabla\cdot\left(\rho_{0}\mathbf{v}_{1}\mathbf{v} _{1}\right) \end{array} $$

where the concatenation of the velocities is meant to denote the dyadic. The continuity condition is

93 $$ \nabla\cdot\mathbf{v}_{2}=-\nabla\cdot\left(\rho_{1}\mathbf{v}_{1}\right) /\rho_{0}  $$

The quantities on the right hand sides of these equations are presumed known. Let us write

94 $$ p_{2}:=p+p^{\prime};\mathbf{v}_{2}:=\mathbf{v}+\mathbf{v}^{\prime}  $$

95 $$ {\kern4pt}p:=K_{C}\ast s  $$

where *K*_*C*_ is the Coulomb kernel, i.e., the free space Green function for the Laplacian. Similarly for **v**, so that these quantities solve the desired equations but do not have the right boundary conditions. Suppose further

96 $$ \left. \mathbf{v}\right\vert_{\partial C}=\mathbf{V}\left(\mathbf{x} \right),\mathbf{x}\in\partial C  $$

Now suppose

97a $$\begin{array}{*{20}l} \eta\Delta\mathbf{v}^{\prime}\mathbf{-}\nabla p^{\prime} & =0 \end{array} $$

97b $$\begin{array}{*{20}l} \nabla\cdot\mathbf{v}^{\prime} & =0 \end{array} $$

97c $$\begin{array}{*{20}l} \left. \mathbf{v}^{\prime}\right\vert_{\partial C} & =-\mathbf{V}\left(\mathbf{x}\right),\mathbf{x}\in\partial C \end{array} $$

Then the pair *p*_2_:=*p*+*p*^′^;**v**_2_:=**v**+**v**^′^ satisfy the equations and the boundary conditions desired (zero velocity at the boundaries). One may then attempt to check if the narrow gaps between obstacles (cells) prevent any microstreaming from developing. We speculate that the dispersivity results in an effective diffusion constant that is likely at least three orders of magnitude smaller than what we ascribed to *D*_*H*_ above, resulting in a negligible effect on spreading particles. Note added A paper [56] came to our attention, thanks to the anonymous reviewer mentioned in the previous endnote. In this paper, the beginnings of a stochastic model for solute transport where the particle is alternately retained by the cell walls or advected by the acoustic wave, are indicated. It is claimed that the resulting model also has a dispersion, or effective diffusion coefficient of the same form as equation (90) though from an entirely different mechanism. However, the paper has several errors: (i) their final equations have the wrong probability as a coefficient multiplying a term due to an algebraic error; (ii) they use approximations that are not *bona fide* expansions – in fact their (invalid) expansion method would obtain a spurious diffusivity from a purely advective flow; (iii) their method correctly has only a purely deterministic limit, which has no transport after averaging; (iv) their purported agreement between simulation and analytic solution may also be an error: if they used the wrong diffusivity for both, they are perhaps comparing a continuum equation with its finite difference discretization, which must agree unless the discretization is poor. Whether their approach can be salvaged requires further study, according to methods described in [57], Sections 3.8.3 and 7.2.3, with quantitative estimates for the effective diffusivity, if any. In any case, if an effective diffusivity due to whatever mechanism is competitive with bulk streaming, it would be even more important to distinguish advective from dispersive effects, as suggested in the last paragraph of our “Conclusions and discussion” section.

### Oscillatory flow dispersion

In the above, we considered dispersive effects that might arise from period-averaged streaming velocities near a boundary. Of course *oscillatory* phenomena will also result in such dispersivity. The relevant other parameter is the period *T* of the oscillation, so that the effective diffusivity from dimensional analysis (and also from a calculation of the mean square displacement of a particle following the same methodology of random distribution of channel directions) is ∼*v*^2^*T* where now *v* is the oscillatory velocity amplitude. Thus, this dispersivity is quadratic in the relevant speed in contrast to the dispersion arising from steady flow which is linear as indicated above. Further, the numerical factor, unavailable from dimensional analysis, approaches $ \frac 12 $ for *A*/*L*>>1, but can be considerably less than that for smaller *A*/*L* (and essentially vanishes as *A*/*L*→1). However, it is obvious that this can only happen when the displacement amplitude *A* is greater than the length of a channel, which is emphatically not the case for any reasonable amplitude for sonication, which has *A*<<1 *μ* m. Otherwise the particle essentially returns along the same channel, which is indeed the case. These oscillatory dispersivities should not pertain to acoustic amplitudes, but may to pulsatile ones due to the heartbeat.

### Dispersion from peristaltic effects

Peristalsis can be a very strong rectification effect of an oscillatory field. By definition, this is absent in the homogenized picture used in our treatment, and further, it depends on transverse oscillations in the boundaries of the pore space. Such peristaltic effects, following the pioneering work summarized in [58] (where earlier work is cited) have been extensively studied and are a very current topic in perivascular flows in the brain. Some work on peristaltic effects of traveling waves includes [59] within a single channel and [60] in a network of channels. Any order of magnitude estimate of such peristalsis due to the small acoustic amplitudes results in a negligible effect. However, we have not undertaken a proof that it cannot be present.

## Conclusions and discussion

Our principal results are the introduction of the basic theory for bulk acoustic streaming in homogenized porous media, and to obtain the Green functions for the streaming equations. We analyzed the porous medium equations in detail to isolate phenomena we feel are the important ones for streaming. We have illustrated the streaming effects in some acoustic fields, showing perhaps suspiciously good agreement with experimental results. We have speculated on the *ineffectiveness* of several effects that depend on the microstructure of cells and extracellular space to transport fluids and particles in acoustic fields; some mechanisms that occur due to attenuation and others that are independent of it. All of these speculations are just that and require further study for verification or refutation.

We emphasize that the basic equations for the streaming in tissue, namely (20a) with 20b) are easily written down for anisotropic inhomogeneous tissue together with fluid loss. We have developed and shown that such equations may be solved for *an individual brain* (and other organs) [32, 61]. The chief difficulty in the present case is to obtain the sources from models of acoustic propagation in tissue. However, as we have mentioned, these models are now quite advanced, driven in particular by applications of high intensity focused ultrasound (HIFU), so we may envisage a combination of these two classes of software models that would allow planning of the proper acoustic fields to effect UeCD and predict its effects on a particular patient. Such a model will need to be supplemented by addressing of safety considerations before any such application becomes reality.

The experimental studies so far have been limited in their usefulness in disentangling the mechanisms for the streaming effects observed. As discussed in the “Conclusions” subsection of the previous section, one serious limitation of the usefulness for clinical application of the experiments so far conducted is that they have not yet exploited the ability of sonication to provide pressure gradients away from the catheter. Also, they have been performed under the conditions of fixed flow rates infused into the medium. It would be useful to conduct the experiments at fixed pressure as well, to observe any effects due to fluid conservation as discussed in the text. The experiments should also quantitate the tracers used, i.e., they should track the concentrations of the tracers. In magnetic resonance imaging, the methods used in [62] could be used, for example. In addition, ideally, the observations should be continuous so that the time advance of the tracer distribution can be studied. Advective or convective effects reveal themselves by showing advance of fronts or mean distributions linear with time, while dispersive and diffusive effects advance in proportion to the square root of time. This difference would show up particularly well in one dimensional systems (e.g., excised tissue in narrow tubes). It would also be useful to compare not merely CED with CED plus sonication (UeCD as we have called it), but sonication alone compared with naturally occurring diffusion. The dispersive effects discussed in the previous section due to microstreaming or peristalsis can be distinguished by varying the frequency of the acoustic excitation since the two mechanisms have very different dependences on the frequency of the ultrasound. Such detailed analyses – which demand knowing the concentration distribution to extract meaningful parameters – will help elucidate the mechanisms involved.

## Appendix 1: Stokes drift and Lagrangian-mean [18] velocities

In addition to the streaming due to attenuation described above, there is also a streaming, independent of attenuation, of fluid particles in a vibratory wave, apparently first described for waves in fluids by Stokes. The way this drift is usually derived is by the venerable Method of Averaging. We use it in the very simple form described in the text by Landau and Lifshitz [63] (the section *Motion in a Rapidly Oscillating Field*). If we assume that the fluid particle is free to move in the sound field, then the position **r**(*t*) of any such particle is obtained by integrating along a trajectory

98 $$ \dot{\mathbf{r}}\left(t\right) \text{ }=\mathbf{v}_{1}\left(\mathbf{r}\left(t\right),t\right)   $$

with an initial condition on **r** which we shall take to be the origin. **v**_1_ is the same as in the text above, and averages to zero. We use the *ansatz*

99 $$ \mathbf{r}=:\mathbf{F}+\mathbf{S}  $$

where **F** and **S** are the fast & slow components, with **F** averaging to zero over a cycle. These are both vectors from an origin, and the idea is that **S** denotes a slow drift superimposed on an oscillatory and small **F**. So,

100 $$ \dot{\mathbf{F}}=\mathbf{v}_{1}\left(\mathbf{S},t\right) \Longrightarrow \mathbf{F}=\int^{t}\mathbf{v}_{1}\left(\mathbf{S},\tau\right) d\tau=:\mathbf{u}\left(\mathbf{S},t\right)  $$

which defines a displacement **u**. Considering this to be small, we get

101 $$ \mathbf{v}_{1}\left(\mathbf{r}\left(t\right),t\right) \approx \mathbf{v}_{1}\left(\mathbf{S},t\right) +\mathbf{u}\cdot\nabla \mathbf{v}_{1}\left(\mathbf{S},t\right)  $$

Averaging Eq. (98), we see that the slow component then satisfies

102 $$ \dot{\mathbf{S}}\left(t\right) =\left\langle \mathbf{u}\cdot\nabla \mathbf{v}_{1}\right\rangle_{T}\equiv\left\langle \mathbf{u}\cdot \nabla\dot{\mathbf{u}}\right\rangle_{T}=:\mathbf{v}_{\operatorname*{Stokes}}   $$

which is an equation involving only the slow variable. We call this **v**_Stokes_ since Stokes was apparently the first to derive it in connection with water waves. Among others, Westervelt derives this for acoustic streaming [19]. In the literature on fluid streaming, it is pointed out that this really should be added to the attenuation–driven streaming velocity **v** (after expressing both in the same reference frame (e.g., spatial coordinates). Furthermore

103 $$ \nabla\cdot\mathbf{v}_{\operatorname*{Stokes}}=\nabla\cdot\mathbf{v}_{0}  $$

where both sides are evaluated to the second order, and **v**_0_ was defined in (6). In other words, the velocities are equal upto a curl. Westervelt [64] shows this, but his proof seems excessively complicated involving expressions with four curls and so on. In case a simpler derivation is useful, we offer one here, though such simplifications must undoubtedly have been presented. We write the equation of continuity to first order:

104 $$ \frac{\partial}{\partial t}\rho_{1}=-\rho_{0}\nabla\cdot\mathbf{v}_{1}  $$

Integrating this, averaging, and keeping terms to second order we see

105 $$ \nabla\cdot\mathbf{v}_{0}\equiv\frac{1}{\rho_{0}}\nabla\cdot\left\langle \left(\rho_{1}\dot{\mathbf{u}}\right) \right\rangle_{T}=-\nabla \cdot\left\langle \left(\left(\nabla\cdot\mathbf{u}\right) \dot {\mathbf{u}}\right) \right\rangle_{T}  $$

We thus have to show that

106 $$ \nabla\cdot\left\langle \dot{\mathbf{u}}\left(\nabla\cdot\mathbf{u}\right) \right\rangle_{T}=\nabla\cdot\left\langle \mathbf{u}\cdot\nabla \dot{\mathbf{u}}\right\rangle_{T}  $$

Taking the del operator inside the average,

107 $$ \nabla\cdot\left\langle \mathbf{u}\cdot\nabla\dot{\mathbf{u}}\right\rangle_{T}=\left\langle \nabla\mathbf{u}:\nabla\dot{\mathbf{u}}\right\rangle_{T}+\left\langle \mathbf{u}\cdot\nabla\left(\nabla\cdot\dot{\mathbf{u} }\right) \right\rangle_{T}   $$

while

108 $$ {\begin{aligned} \nabla\!\cdot\!\left\langle \dot{\mathbf{u}}\left(\nabla\!\cdot\!\mathbf{u}\right) \right\rangle_{T}\,=\,\left\langle \left(\nabla\cdot\mathbf{u}\right) \left(\nabla\cdot\dot{\mathbf{u}}\right) \right\rangle_{T}\,+\,\left\langle \mathbf{u}\cdot\nabla\left(\nabla\cdot\dot{\mathbf{u}}\right) \right\rangle_{T}  \end{aligned}}  $$

(It is easier and immediate to get this by decomposing into Cartesian components). However the first term within the time average on the right hand side of (108) is just *∂*((∇·**u**)^2^)/*∂t* while the corresponding term in (107) is *∂*(∇**u**:∇**u**)/*∂t*. Both of these being time derivatives vanish upon averaging over a period of the wave, and thus the two velocities indeed have the same divergence, and so are equal upto the curl of a vector field.

However, in the case of porous media, we are always within the inner boundary layer as we have stated. The fluid particle are as little free to drift as the solid particles and this effect should be negligible in a porous medium. We now show this. We will be rather pedantic in our notation, to avoid ambiguity, and first obtain a well-known relation between the most natural definition of a material or Lagrangian velocity and an Eulerian velocity at a different location [65]. We present this derivation only to introduce our notation. Then, the next subsection argues for neglect of Stokes drift in a porous medium.

Let

109 $$ \mathbf{r}=\overrightarrow{f}\left(t|\mathbf{X},s\right)  $$

be the position of a “particle” at time *t*, given that it was at **X** at time *s*. $\overrightarrow {f}$ is supposed to be a diffeomorphism of three dimensional space (the Jacobian is non-singular). The forward arrow indicates that we are dealing with a function of the material coordinates. The Lagrangian velocity is defined as

110 $$ \overrightarrow{\mathbf{v}}=\frac{\partial}{\partial t}\overrightarrow{f} \left(t|\mathbf{X},s\right)  $$

indicating clearly that **X**,*s* are to be held fixed. We have the basic identity

111 $$ \overrightarrow{f}\left(t|\mathbf{X},s\right) =\overrightarrow{f}\left(t|\overrightarrow{f}\left(s+ds|\mathbf{X},s\right),s+ds\right)  $$

for a flow, so that by differentiation we write down a condition of invariance with respect to a change of labeling time:

112 $$ \frac{\partial}{\partial s}\overrightarrow{f}\left(t|\mathbf{X},s\right) +\frac{\partial}{\partial s}\overrightarrow{f}\left(s|\mathbf{X},s\right) \cdot\nabla\overrightarrow{f}\left(t|\mathbf{X},s\right) =0   $$

Since there is only one set of variables in space (**X**), it should be clearly understood that the gradient is taken with respect to this set. However, the second term in the equation in fact is an Eulerian velocity, which we denote

113 $$ \overleftarrow{\mathbf{v}}\left(\mathbf{X},s\right) :=\frac{\partial }{\partial s}\overrightarrow{f}\left(s|\mathbf{X},s\right)  $$

Note that Eq. (112) could be written for *any* quantity (not just $\overrightarrow {f}$), and says that quantities are conserved along the characteristics obtained by integrating the Eulerian velocity along its trajectory. We therefore write the same equation for the Lagrangian velocity:

114 $$ \frac{\partial}{\partial s}\overrightarrow{\mathbf{v}}\left(t|\mathbf{X},s\right) +\overleftarrow{\mathbf{v}}\left(\mathbf{X},s\right) \cdot \nabla\overrightarrow{\mathbf{v}}\left(t|\mathbf{X},s\right) =0   $$

Integrating we obtain

115 $$ \overrightarrow{\mathbf{v}}\left(t|\mathbf{X},s\right) -\overleftarrow{\mathbf{v}}\left(\mathbf{X},t\right) =\int_{s} ^{t}\overleftarrow{\mathbf{v}}\left(\mathbf{X},t^{\prime}\right) \cdot\nabla\overrightarrow{\mathbf{v}}\left(t|\mathbf{X},t^{\prime}\right) dt^{\prime}   $$

The left hand side involves two different functions and the first term on the right involves the Eulerian velocity at the *current* time at the spatial position where the fluid particles were *originally* labeled. Averaging over an acoustic cycle gives us the difference between the average Lagrangian and Eulerian velocities.

### Absence of Stokes drift in porous media

In this subsection, and only in this subsection, we define the symbol **w** as

116 $$ \mathbf{w}:=\mathbf{v}^{f}-\mathbf{v}^{s}  $$

where the superscripts *f*,*s* refer to fluid and solid, respectively. (In the main sections, **w** is defined to be the pore or interstitial volume fraction times the relative velocity). The decorations on **w** will be inherited in an obvious way from those on the **v**^′^s. Then we see that applying (114) in turn to the fluid and the solid and subtracting yields

117 $$ \begin{aligned}\frac{\partial}{\partial s}\overrightarrow{\mathbf{w}}\left(t|\mathbf{X},s\right) &+\overleftarrow{\mathbf{w}}\left(\mathbf{X},s\right) \cdot \nabla\overrightarrow{\mathbf{w}}\left(t|\mathbf{X},s\right) \\&+\overleftarrow{\mathbf{v}}^{s}\left(\mathbf{X},s\right) \cdot \nabla\overrightarrow{\mathbf{w}}\left(t|\mathbf{X},s\right) \\&+\overleftarrow{\mathbf{w}}\left(\mathbf{X},s\right) \cdot\nabla \overrightarrow{\mathbf{v}}^{s}\left(t|\mathbf{X},s\right) =0 \end{aligned}  $$

Hence

118 $$ {}\begin{aligned} \overrightarrow{\mathbf{w}}\left(t|\mathbf{X},s\right) -\overleftarrow{\mathbf{w}}\left(\mathbf{X},t\right) & =\int_{s}^{t}dt^{\prime}\overleftarrow{\mathbf{w}}\left(\mathbf{X},t^{\prime}\right) \cdot\nabla\overrightarrow{\mathbf{w}}\left(t|\mathbf{X},t^{\prime}\right) \\ & \quad +\int_{s}^{t}dt^{\prime} \overleftarrow{\mathbf{v}}^{s}\left(\mathbf{X},t^{\prime}\right) \cdot\nabla\overrightarrow{\mathbf{w}}\left(t|\mathbf{X},t^{\prime}\right)\\ &\quad +\int_{s}^{t}dt^{\prime}\overleftarrow{\mathbf{w}}\left(\mathbf{X},t^{\prime}\right) \cdot\nabla\overrightarrow{\mathbf{v}}^{s}\left(t|\mathbf{X},t^{\prime}\right)  \end{aligned}  $$

As before, we now expand

119 $$ \mathbf{w}=\mathbf{w}_{1}+\mathbf{w}_{2}  $$

both with and without decorations. However, upto and including second order,

120 $$ \mathbf{v}^{s}=\mathbf{v}_{1}^{s}  $$

there being no net drift of the solid. The first order quantities are of course the same in the Eulerian and Lagrangian viewpoints. Let us substitute these into (118) and average over a cycle, retaining only upto the second order quantities. The result is:

121 $$\begin{array}{*{20}l} & \overrightarrow{\mathbf{w}}_{2}\left(t|\mathbf{X},s\right) -\overleftarrow{\mathbf{w}}_{2}\left(\mathbf{X},t\right)  \end{array} $$

122 $$\begin{array}{*{20}l} & =\int_{s}^{t}dt^{\prime}\left\langle \overleftarrow{\mathbf{w}}_{1}\left(\mathbf{X},t^{\prime}\right) \cdot\nabla\overrightarrow{\mathbf{w}} _{1}\left(t|\mathbf{X},t^{\prime}\right) \right\rangle \\ & \quad +\int_{s} ^{t}dt^{\prime}\left\langle \overleftarrow{\mathbf{v}}_{1}^{s}\left(\mathbf{X},t^{\prime}\right) \cdot\nabla\overrightarrow{\mathbf{w}} _{1}\left(t|\mathbf{X},t^{\prime}\right) \right\rangle \\ & \quad+\int_{s}^{t}dt^{\prime}\left\langle \overleftarrow{\mathbf{w}}_{1}\left(\mathbf{X},t^{\prime}\right) \cdot\nabla\overrightarrow{\mathbf{v}}_{1} ^{s}\left(t|\mathbf{X},t^{\prime}\right) \right\rangle \end{array} $$

Now $\overrightarrow {\mathbf {w}}_{1}=$$\overleftarrow {\mathbf {w}}_{1}$, (to first order the Eulerian and Lagrangian velocities are the same), and so the first of the three terms should vanish since the dot product involves two quantities out of phase with each other. Further, with our “Imaging Community assumption” for the acoustic waves, $\overrightarrow {\mathbf {w}}_{1}$ vanishes and so all the terms on the right hand side of (121) do as well. Hence there will be no Stokes drift in the context of homogenized porous media. A more accurate treatment would follow the transition from the boundary layer behavior of the fluid particles to the behavior in bulk according to the frequency of the wave and the interstitial widths.

## Appendix 2: Green’s functions for the streaming equations

We here ‘derive’ the Green functions given in Eq. (132) below. Let us introduce a parameter *α* with dimensions of inverse length

123 $$ \alpha:=+\sqrt{\frac{\gamma}{\eta}}   $$

The usual method for obtaining Green’s functions is to use the Fourier transform. With $\widehat {\mathbf {k}}=\mathbf {k}/k$ denoting the unit vector in the **k** direction, (31a) and (31b) give

124 $$ \eta\left(\alpha^{2}+k^{2}\right) \widetilde{\mathbf{v}} =\widetilde{\mathbf{f}}-i\mathbf{k}\widetilde{p},\text{ \ \ \ }\mathbf{k\cdot }\widetilde{\mathbf{v}}\mathbf{=-k\cdot}\widetilde{\mathbf{v}}_{0}  $$

where the tildes of course denote the Fourier transforms of the quantities without the tilde. Taking the dot product with *k* gives

125 $$ \eta\left(\alpha^{2}+k^{2}\right) \mathbf{k\cdot}\widetilde{\mathbf{v}} _{0}+\widetilde{\mathbf{f}}\cdot\mathbf{k}=ik^{2}\widetilde{p}  $$

which allows us to substitute for the Fourier transform of the pressure, and so we obtain

126 $$ \widetilde{\mathbf{v}}=\frac{\widetilde{\mathbf{f}}-\left(\widetilde{\mathbf{f}}\cdot\widehat{\mathbf{k}}\right) \widehat{\mathbf{k}} }{\eta\left(\alpha^{2}+k^{2}\right) }-\left(\widetilde{\mathbf{v}} _{0}\cdot\widehat{\mathbf{k}}\right) \widehat{\mathbf{k}}  $$

The inverse transform would give us the desired solution, but we give here a simple and direct though hand-waving derivation for the Green function. The result can be checked directly as well. We note that

127 $$ \nabla\frac{1}{\left\vert \mathbf{x}-\mathbf{x}^{\prime}\right\vert } =-\nabla^{\prime}\frac{1}{\left\vert \mathbf{x}-\mathbf{x}^{\prime}\right\vert }  $$

(The prime denotes of course the gradient with respect to the **x**^′^ variables). We may therefore take the gradient of equation (34), integrate twice by parts, discarding the boundary terms in each case (so that the sources are such that the terms must vanish at infinity) to obtain (recall the notation as mentioned in the Introduction)

128 $$ -4\pi\nabla p=\left(\left[ \gamma-\eta\nabla^{2}\right] \mathbf{v} _{0}+\mathbf{f}\right) \ast\cdot\text{ }\nabla\nabla\frac{1}{r}   $$

We substitute (128) into (31a),

129 $$ {}-4\pi\eta\nabla^{2}\mathbf{v+}4\pi\gamma\mathbf{v}=\left(\left[ \gamma -\eta\nabla^{2}\right] \mathbf{v}_{0}+\mathbf{f}\right) \ast\cdot\text{ }\nabla\nabla\frac{1}{r}+4\pi\mathbf{f}\ast\delta  $$

where we write **f=f**∗*δ*, the convolution with the delta distribution. We factor *η* out of the expressions, introducing the *α* of Eq. (123). We may now write the inverse of the “Yukawa operator” (which is defined to be ∇^2^− *α*^2^) acting on the Green function 1/*r* as

130 $$ \left(\nabla^{2}-\alpha^{2}\right)^{-1}1/r=-\frac{1-\exp\left(-\alpha r\right) }{\alpha^{2}r}   $$

We have obtained (130) by solving for (∇^2^−*α*^2^)*h*(*r*)=1/*r*, discarding one constant of integration by demanding the solution vanish at infinity, and fixing the other constant by demanding agreement with the Green function for the Yukawa operator, i.e., we set

131 $$ -4\pi\left(\nabla^{2}-\alpha^{2}\right)^{-1}\delta^{3}\left(\mathbf{x}\right) =\exp\left(-\alpha r\right) /r   $$

Thus if we apply the Laplace operator to *h*(*r*), it is equivalent to applying (∇^2^−*α*^2^)^−1^ to − 4*πδ*^3^(**x**) which should yield the Yukawa potential of (131). This fixes the other constant of integration to be 1/*α*^2^. Returning to (129), we apply (∇^2^−*α*^2^)^−1^ to both sides, and use (130) and (131), to find

132 $$ {}\begin{aligned} 4\pi\gamma\mathbf{v}&=\gamma\mathbf{v}_{0}\ast\cdot\text{ }\nabla\nabla\frac {1}{r}+\mathbf{f}\ast\cdot\text{ }\nabla\nabla\frac{1-\exp\left(-\alpha r\right) }{r}\\ & \quad +\alpha^{2}\mathbf{f}\ast\exp\left(-\alpha r\right) /r  \end{aligned}  $$

Further expansion of the application of ∇∇ to $\frac {1}{r}$ and to $\frac {\exp \left (-\alpha r\right) }{r}$is given in the text, in the “Green’s functions for the streaming equations” section.

### Bulk fluid limit

In a pure fluid, *γ*→0, but we must expand to non-vanishing powers of *α*, using Eq. (38), dividing first by *γ* and then replacing $\frac {\alpha ^{2}}{\gamma }$ by the viscosity *η*:

133 $$ {}\begin{aligned} & 4\pi\mathbf{v}\left(\mathbf{r}\right) \overset{\gamma\rightarrow 0}{\underset{\alpha\rightarrow0}{\longrightarrow}}\mathbf{f}\left(\mathbf{r}\right) \mathbf{\ast}\left\{ \frac{\alpha^{2}}{\gamma r} +\frac{\alpha\left(1-\alpha r\right) }{\gamma r^{2}}+\frac{1-\alpha r+\left(\alpha r\right)^{2}/2}{\gamma r^{3}}-\frac{1}{\gamma r^{3} }\right\} \\ & +\mathbf{f}\left(\mathbf{r}\right) \mathbf{\ast\cdot}\left\{ \frac{\widehat{\mathbf{r}}\widehat{\mathbf{r}}}{r}\left(\frac{3}{\gamma r^{2}}-\frac{\alpha^{2}}{\gamma}-\frac{\alpha\left(1-\alpha r\right) }{\gamma r}-3\frac{1-\alpha r+\left(\alpha r\right)^{2}/2}{\gamma r^{2} }\right) \right\} \\ & -\mathbf{v}_{0}\left(\mathbf{r}\right) \ast\mathbf{\cdot}\left\{ \frac{\mathbb{I}-3\widehat{\mathbf{r}}\widehat{\mathbf{r}}}{r^{3}}\right\} -4\pi\mathbf{v}_{0}\left(\mathbf{r}\right) \end{aligned}  $$

Thus

134 $$ {\begin{aligned} \mathbf{v}\left(\mathbf{r}\right) \overset{\gamma\rightarrow 0}{\underset{\alpha\rightarrow0}{\longrightarrow}}\frac{\mathbf{f}\left(\mathbf{r}\right) }{8\pi\eta}\mathbf{\ast\cdot}\left\{ \frac{\mathbb{I} +\widehat{\mathbf{r}}\widehat{\mathbf{r}}}{r}\right\} +\frac{\mathbf{v} _{0}\left(\mathbf{r}\right) }{4\pi}\ast\mathbf{\cdot}\left\{ \frac{3\widehat{\mathbf{r}}\widehat{\mathbf{r}}-\mathbb{I}}{r^{3}}\right\} -\mathbf{v}_{0}\left(\mathbf{r}\right) \end{aligned}}  $$

The standard result [6] which is the usual “Stokeslet” familiar from the fluid dynamics of slow viscous flow is the first term on the right. The second term arises from the mass conservation forcing and as Lighthill points out, decreases like *r*^−3^ unlike the first. There is a third term as well from the Green function singularity. Lighthill also remarks that such Green functions are of limited value in discussing streaming in pure fluids owing to the importance of including the convective acceleration term in the equations. Then the equations become nonlinear and of course there are no Green function or linear superposition results available. Remarkably, there is an exact solution for the pure fluid problem with a point source, due to Landau and Squire: see the quoted paper of Lighthill for details. As described in the “Terms neglected” subsection, this nonlinear term is of no importance in the porous medium.

### Darcy (porous medium) limit

The *η*→0, *α*→*∞* limit defines the porous medium. From (132), we get immediately

135 $$ 4\pi\gamma\mathbf{v}=\left(\gamma\mathbf{v}_{0}+\mathbf{f}\right) \ast \cdot\text{ }\nabla\nabla\frac{1}{r}+\operatorname*{Lim}_{\alpha \rightarrow\infty}\alpha^{2}\mathbf{f}\ast\exp\left(-\alpha r\right) /r  $$

Now, Lim_*α*→*∞*_*α*^2^*e*^−*αr*^=−*δ*^′^(*r*), the prime denoting a derivative with respect to the argument. Thus the second term evaluates (including the integration with respect to the angles and using the meaning of the derivative of the Dirac delta) to

136 $$ \left. 4\pi\frac{d}{dr^{\prime}}\left(r^{\prime}\mathbf{f}\left(\mathbf{x}-\mathbf{x}^{\prime}\right) \right) \right\vert_{r^{\prime} =0}=4\pi\mathbf{f}\left(\mathbf{x}\right)  $$

provided $\operatorname *{Lim}_{r^{\prime }\rightarrow 0}$$r^{\prime } \frac {\partial }{\partial r^{\prime }}\mathbf {f}\left (\mathbf {x} -\mathbf {x}^{\prime }\right) =0$. Thus

137 $$ \gamma\mathbf{v}=\frac{1}{4\pi}\left(\gamma\mathbf{v}_{0}+\mathbf{f}\right) \ast\cdot\text{ }\nabla\nabla\frac{1}{r}+\mathbf{f}  $$

This result is also shown starting directly from the porous medium equation in the “Green’s functions for the streaming equations” section.

## Abbreviations

CED: Convection-enhanced delivery
UeCD: Ultrasound-enhanced convective delivery

## Footnotes

1 This identity has a rather checkered history. Referring to the regular and singular parts, even more recently than sixty years ago (see e.g., the first edition of Jackson’s classic text, now in its third [22]), the singular part would be routinely omitted despite the fact that it was well known that the *Laplacian* of 1/r is a distribution (Dirac delta). The first volume of the equally classic treatise [23] appeared in English only in 1964. In that, Gelfand and Shilov show clearly how such identities should be treated and their singular parts extracted. The latter involves integrating an expression over a surface enclosing the singularity. Unfortunately, they proceed to illustrate this with the isotropic Laplacian operator, where the surface is chosen to be a sphere, and the result well known. Almost two full decades after this hardly obscure book had been available, Frahm rediscovered this amongst other delta function identities [24]. His result for the dyadic ∇∇(1/*r*) is entirely equivalent to the Gelfand-Shilov method when using a spherical surface for the regularization, and he obtained − 4*π**δ*^(3)^(**x**)/3 for the singular part. Jackson in subsequent editions gives this expression as well, with some “physicist’s arguments” for it. Finally, Franklin – in the citation referenced – produced the identity we use. We may also derive it by Gelfand’s method choosing an ellipsoidal surface for the regularization procedure. In fact the isotropic singular expression of Jackson and Frahm would give incorrect results for the spherical source; see “The Green function singularity” subsection.

2 Model RK 100: http://www.fusinstruments.com/products/lp-100/.

3 We accounted for the 20−fold higher concentration of the viscosity of the contrast agent used (Visipaque 320™) relative to that of water in the calculation. (The hydraulic conductivity is reduced by this factor). This makes no difference to the theoretical results as noted above. We did not, however, alter the attenuation coefficient of the sound wave in the calculation.

4 We thank an anonymous referee for this more recent reference which does not allude to the earlier.
